# Trip13 Depletion in Liver Cancer Induces a Lipogenic Response Contributing to Plin2‐Dependent Mitotic Cell Death

**DOI:** 10.1002/advs.202104291

**Published:** 2022-08-28

**Authors:** Marcos Rios Garcia, Bettina Meissburger, Jessica Chan, Roldan M. de Guia, Frits Mattijssen, Stephanie Roessler, Andreas L. Birkenfeld, Nathanael Raschzok, Fabien Riols, Janina Tokarz, Maude Giroud, Manuel Gil Lozano, Goetz Hartleben, Peter Nawroth, Mark Haid, Miguel López, Stephan Herzig, Mauricio Berriel Diaz

**Affiliations:** ^1^ Institute for Diabetes and Cancer Helmholtz Center Munich 85764 Neuherberg Germany; ^2^ Joint Heidelberg‐IDC Translational Diabetes Program Inner Medicine 1 University Hospital 69120 Heidelberg Germany; ^3^ German Center for Diabetes Research (DZD) 85764 Neuherberg Germany; ^4^ NeurObesity Group Department of Physiology CIMUS University of Santiago de Compostela‐Instituto de Investigación Sanitaria Santiago de Compostela 15782 Spain; ^5^ Joint Division Molecular Metabolic Control DKFZ‐ZMBH Alliance and Network Aging Research German Cancer Research Center (DKFZ) 69120 Heidelberg Germany; ^6^ Institute of Pathology Liver Cancer Center Heidelberg (LCCH) University Hospital Heidelberg Im Neuenheimer Feld 224 69120 Heidelberg Germany; ^7^ Institute of Diabetes Research and Metabolic Diseases (IDM) of the Helmholtz Center Munich 72076 Tübingen Germany; ^8^ Department of Diabetes School of Life Course Science King's College London London WC2R 2LS UK; ^9^ Department of Surgery Campus Charité Mitte and Campus Virchow‐Klinikum Charité‐Universitätsmedizin Berlin 2, Berlin Institute of Health (BIH) 10117 Berlin Germany; ^10^ Metabolomics and Proteomics Core Helmholtz Center Munich 85764 Neuherberg Germany; ^11^ Department of Medicine 1 and Clinical Chemistry University of Heidelberg Im Neuenheimer Feld 410 69120 Heidelberg Germany; ^12^ CIBER Fisiopatología de la Obesidad y Nutrición (CIBEROBN) Santiago de Compostela 15706 Spain; ^13^ Chair Molecular Metabolic Control Technical University Munich Germany

**Keywords:** hepatocellular carcinoma, lipogenesis, mitosis, MTOCs, Plin2, spindle assembly checkpoint, Trip13

## Abstract

Aberrant energy metabolism and cell cycle regulation both critically contribute to malignant cell growth and both processes represent targets for anticancer therapy. It is shown here that depletion of the AAA+‐ATPase thyroid hormone receptor interacting protein 13 (Trip13) results in mitotic cell death through a combined mechanism linking lipid metabolism to aberrant mitosis. Diminished Trip13 levels in hepatocellular carcinoma cells result in insulin‐receptor‐/Akt‐pathway‐dependent accumulation of lipid droplets, which act as functional acentriolar microtubule organizing centers disturbing mitotic spindle polarity. Specifically, the lipid‐droplet‐coating protein perilipin 2 (Plin2) is required for multipolar spindle formation, induction of DNA damage, and mitotic cell death. Plin2 expression in different tumor cells confers susceptibility to cell death induced by Trip13 depletion as well as treatment with paclitaxel, a spindle‐interfering drug commonly used against different cancers. Thus, assessment of Plin2 levels enables the stratification of tumor responsiveness to mitosis‐targeting drugs, including clinically approved paclitaxel and Trip13 inhibitors currently under development.

## Graphical abstract

A novel mechanism is presented by which depletion of the ATPase thyroid hormone receptor interacting protein 13 (Trip13) results in mitotic cell death, including enhanced lipogenesis and a role of lipid droplet accumulation in aberrant mitotic spindle polarity. Specifically, perilipin 2 confers susceptibility to Trip13‐knockdown‐ and antimitotic‐drug‐induced cell death, allowing for stratification for paclitaxel‐treatment‐responsive tumor cells.

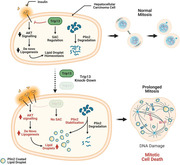

## 1 Introduction

Cancer is characterized by sustained cell proliferation with high demands for cellular energy and building blocks which require specific metabolic adaptations, commonly referred to as metabolic reprograming.^[^ ^1^ ^]^ However, despite the fact that altered metabolism is now considered an important hallmark of cancer, the role of specific metabolic pathways, such as “de novo” fatty acid and lipid synthesis as well as storage in lipid droplets, is far from being fully elucidated,^[^ ^2^ ^]^ and may even be regulated in a context‐ and tumor‐type‐specific manner.^[^ ^3^ ^]^

In addition, malignant cell proliferation is associated with alterations in cell cycle control, which in turn is interrelated with other central features of tumor development, such as chromosomal instability and aneuploidy.^[^ ^4^ ^]^ Mitosis is a cell cycle related, tightly controlled process with high energy demands.^[^ ^5^ ^]^ Conceptually, it is reasonable to assume that functional connections must exist between regulation of metabolism and cell cycle control. Still, if and how metabolic regulation is associated with mitosis and particularly mitotic spindle abnormalities as well as genomic instability in malignant cells is widely unexplored.

During normal mitosis, centrosomes regulate cell bipolarity and chromosome alignment in the metaphase plane. This alignment is a prerequisite for the inactivation of the spindle assembly checkpoint (SAC) through disassembly of its key effector, the mitotic checkpoint complex (MCC).^[^ ^6^ ^]^ Different aberrations in the mitotic spindle formation induce persistent SAC activation and mitotic arrest, which can result in mitotic cell death. In this context, it is important to note that escape from mitotic arrest might underlie the development of resistance to antimitotic drugs limiting their efficacy in cancer therapy, a process still incompletely understood.^[^ ^7^ ^]^

Microtubules are core constituents of the mitotic spindle, and arise from discrete sites of microtubule nucleation, termed microtubule organizing centers (MTOCs).^[^ ^8^ ^]^ Historically, MTOC function has been assigned exclusively to centrosomes,^[^ ^9^ ^]^ but recent studies have established that nondividing cells often develop acentriolar (a) MTOCs.^[^ ^10^ ^]^ These aMTOCs were found to form at various locations throughout the cytoplasm and, in contrast to centrosome MTOCs, generate a dynamic and unoriented microtubule array.^[^ ^10^ ^]^ These findings suggest a functional role for aMTOCs in nonmitotic processes, such as cell polarity, migration, invasion, and intracellular trafficking.^[^ ^11^ ^]^

The AAA+ family ATPase thyroid hormone receptor interacting protein 13 (Trip13) has initially been characterized concerning its role in chromosome recombination during meiosis.^[^ ^12^ ^]^ Subsequently, Trip13 was found to play an essential role in p31^comet^‐dependent MCC silencing during mitosis,^[^ ^13^ ^]^ allowing for p31^comet^‐binding to mitotic arrest deficient 2 (Mad2) and causing MCC disassembly, enabling the cell division cycle protein 20 homolog (Cdc20) release, anaphase‐promoting complex (APC/C) activation, and metaphase to anaphase progression.^[^ ^14^ ^]^ When in its open conformation, Mad2 can bind target proteins that contain Mad2 interacting motifs (MIMs) such as Cdc20. Thus, Mad2 in open conformation is mandatory to be recruited to the MCC and activate the SAC. In this state, Mad2 in closed conformation stabilizes the MCC. Trip13 has been described as a key protein in this process and its activity is required to reopen the Mad2 for MCC disassembly. It has been described that in cells lacking Trip13 function, Mad2 remains in the closed conformation, preventing the binding to MIM containing proteins.^[^ ^15^ ^]^ Interestingly, elevated levels of Trip13 have been shown for various types of cancer,^[^ ^16^ ^]^ and in accordance with a function in the control chromosome segregation, are associated with chromosomal instability gene signatures.^[^ ^16^, ^17^ ^]^

Our study now discovers a new role of Trip13 in metabolic reprograming in hepatocellular carcinoma (HCC). We show that Trip13 expression is elevated in both murine and human HCC. Depletion of Trip13 induced insulin‐receptor‐/Akt‐pathway‐dependent lipogenesis. Upon diminished Trip13 levels, lipid droplets accumulated and acted as functional aMTOCs disturbing spindle polarity during mitosis, which triggered tumor cell death. This new role of lipid droplets in mitosis was dependent on perilipin2, and the described combinatorial effects of Trip13 depletion identify an undescribed mechanism of mitotic cell death. Of clinical relevance, we demonstrate that Plin2 expression in different tumor cells confers susceptibility to Trip13‐depletion‐induced mitotic cell death and, importantly, to paclitaxel treatment, the latter representing a mitotic‐spindle‐interfering chemotherapeutic drug commonly used against different cancers. Following the concept of precision medicine, assessing Plin2 levels will allow for the stratification of tumor responsiveness to specific mitosis‐targeting drugs, including future Trip13 inhibitors as well as clinically established drugs such as paclitaxel.

## 2 Results

### 2.1 Trip13 Is Induced in Human and Mouse Hepatocellular Carcinoma

**Figure 1:**
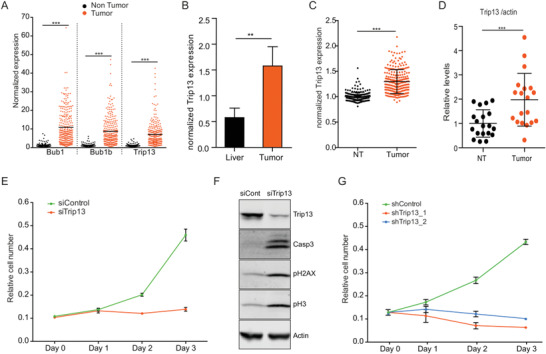
Trip13 is induced in human and mouse hepatocellular carcinoma. A) Bub1, Bub1b, and Trip13 mRNA expression levels in human liver samples from publically available data (E‐GEOD‐25097; https://www.ebi.ac.uk/arrayexpress). B) Quantification of Trip13 mRNA levels in normal mouse liver or liver tumors induced by dimethylnitrosamine (DEN) treatment. C) Trip13 mRNA expression levels (log2) in nonmalignant human liver tissue, NT (*n* = 239), or matched hepatocellular carcinoma, tumor (*n* = 247). D) Quantification of Trip13 protein levels in nonmalignant human liver tissue, N, or matched hepatocellular carcinoma, T (*n* = 19). E) Growth curve of HLF cells transfected with siRNA control or siTrip13 (*n* = 3). F) Western blot cells from (E) showing levels of Trip13, cleaved caspase‐3, and pHistone H3 (*n* = 3). G) Growth curve of HLF cells infected with control shRNA or two different Trip13‐targeting shRNAs (*n* = 3). All data in the figure are shown as the mean ± SEM. *n* numbers refer to biological replicates. (A, B, C, D) Student's *t*‐test. *p < 0.05, **p < 0.01, ***p < 0.001.

We used publicly available expression data on healthy human liver and hepatocellular carcinoma (E‐GEOD‐25097) to study deregulated mitotic proteins. Interestingly, we observed that the top three upregulated mitotic proteins in human HCC were involved in the SAC (Bub1, Bub1b, and Trip13) (**Figure** **1**A). Intriguingly, Trip13 was also upregulated at messenger RNA (mRNA) and protein levels in tumor tissue from a diethylnitrosamine (DEN)‐induced mouse model of liver cancer (Figure 1B and Figure S1A (Supporting Information)). Confirming our animal data and previous studies,^[^ ^16b,e^ ^]^ the expression levels of Trip13 were upregulated in an independent set of human HCC and matched normal livers (Figure 1C). As an additional independent confirmation, we also determined Trip13 protein levels in 19 HCC and the corresponding normal liver samples. Trip13 protein levels were consistently increased in tumor tissue compared to nontumor liver tissue from the same patients (Figure 1D and Figure S1B (Supporting Information)), demonstrating that the SAC‐regulating^[^ ^18^ ^]^ Trip13 represents a highly deregulated component in the pathophysiology of human and mouse HCC.

In line with its proposed role as a therapeutic target in various tumor entities,^[^ ^19^ ^]^ Trip13 knockdown (KD) reduced HCC cell number in vitro with increased apoptosis and DNA damage (Figure 1E,F). Interestingly, we also observed an increase in phospho‐histone H3 serine 10, a marker of cells in mitosis (Figure 1F). Next, by using lentiviral‐mediated small hairpin RNA (shRNA) delivery, we targeted mouse and human Trip13 in several HCC cell lines (Hepa1‐6, HLF, and Huh7). In agreement with previous findings from different cancer cells,^[^ ^16^ ^]^ Trip13 KD was sufficient to completely blunt HCC cell proliferation in vitro (Figure 1G and Figure S1C–E (Supporting Information)). To further validate these findings, we investigated the effect of Trip13 KD in HCC cell lines in vivo by injecting control and Trip13 KD cells subcutaneously into mice. KD of Trip13 in Hepa1‐6 cells resulted in markedly smaller tumors compared to tumors formed upon injection of control cells (Figure S1F,G, Supporting Information). Notably, Huh7 and HLF cells completely lost their tumorigenic capacity upon Trip13 KD (Figure S1H, Supporting Information). Together, these data supported the notion that Trip13 plays an important role in maintaining oncogenic properties in HCC cells in vitro and in vivo.

### 2.2 Loss of Trip13 Triggers Mitotic Failure to Induce Cell Death

**Figure 2:**
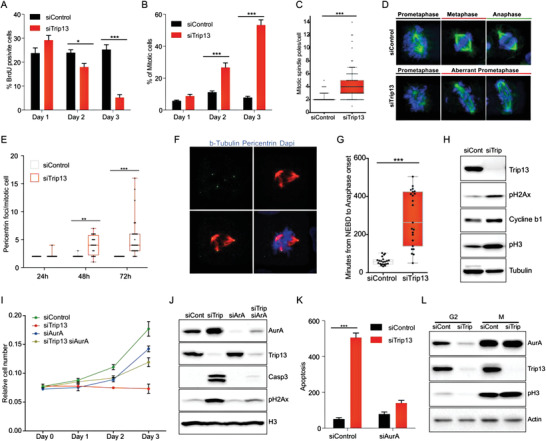
Loss of Trip13 triggers mitotic failure to induce cell death. A) BrdU incorporation at days 1, 2, or 3, post‐transfection in HLF cells transfected with control or Trip13‐targeting siRNA (*n* = 3). B) Mitotic index in HLF cells at days 1, 2, or 3 after siControl or siTrip13 transfection. C) Number of mitotic poles per cell in control or Trip13 KD HLF cells. Cells were IF stained for tubulin and spindle poles were counted (*n* = 3). D) Representative immunofluorescence (IF) images of HLF cells showing mitotic shapes at day 3 after Trip13 KD. Green (tubulin), blue (DAPI) (*n* = 3). E) Quantification of pericentrin IF foci per mitotic cell in HLF control or Trip13 KD cells (*n* = 3). F) Representative images showing pericentrin foci in Trip13 KD HLF cells. G) HLF cells expressing H2B‐Cherry and transfected with siControl or siTrip13 were followed during mitosis by live microscopy from the time of nuclear envelope breakdown (NEBD) to anaphase (*n* = 2). H) Protein extracts from control or Trip13 KD HLF cells were used for pH2Ax, pH3, and Cyclin b1 western blot analysis (*n* = 6). I) Growth curve of HLF cells transfected with siControl, siTrip13, siAurora kinase A, or combinations (*n* = 3). J) Protein levels of cleaved caspase 3 and pH2Ax in HLF cells transfected as in (I) (*n* = 3). K) Apoptosis measured by caspase 3/7 activity of HLF cells transfected with siControl, siTrip13, siAurora kinase A, or siTrip13 and siAurora kinase A (*n* = 2). L) Western blot of siControl‐ or siTrip13‐transfected HLF cells after synchronization in G2 or mitosis, showing levels of Aurora kinase A (*n* = 3). All data in the figure are shown as the mean ± SEM. *n* numbers refer to biological replicates. (A, B, E, K) 1‐way ANOVA with Tukey's Multiple Comparison test. (C, G) Student's *t*‐test. *p < 0.05, **p < 0.01, ***p < 0.001.

To further characterize the mechanisms involved in Trip13 KD effects in liver cancer cells, we focused on HLF cells as a representative human HCC cell line. Bromodeoxyuridine (BrdU) incorporation was markedly reduced at day three post‐transfection with Trip13–small interfering RNA (siRNA) in parallel with an increased level of apoptosis (**Figure** **2**A and Figure S2A (Supporting Information)). Interestingly and in agreement with the elevated levels of phospho‐histone H3 (pH3), we observed a gradual increase in the mitotic index upon Trip13 KD, suggesting a prolongation of the time the cells remain in mitosis (Figure 2B). To investigate the process of mitosis in more detail in our panel of HCC cells, we performed immunofluorescence (IF) staining for tubulin, showing drastically disturbed spindle orientation in Trip13 KD cells, characterized by chromosome mislocalization with no metaphase plate formation and an increased number of mitotic poles (Figure 2C,D). Next, we stained for pericentrin which acts as a scaffold protein exerting a crucial role in MTOC formation. Indeed, quantification of pericentrin foci confirmed the formation of multiple MTOCs per cell, with gradually increasing number from day one to day three post‐transfection of Trip13–siRNA (Figure 2E,F). To further confirm these findings, we performed live imaging of HLF cells stably expressing histone H2B‐mCherry, the latter enabling a sensitive analysis of chromosome dynamics, and determined the time of mitosis. In agreement with our previous data, KD of Trip13 resulted in an approximately fourfold prolongation of the time of mitosis (Figure 2G). Interestingly, most of the Trip13 KD cells died during mitosis with only few cells progressing to G1 phase (data not shown). Consistent with live imaging data, western blot (WB) analysis revealed elevated levels of pH3 and cyclin B1 in Trip13 KD cells compared to control cells, the accumulation of which under conditions of diminished cell proliferation was indicative of mitotic delay (Figure 2H). DNA damage determined by phospho‐histone H2Ax (pH2Ax) was also increased in Trip13 KD cells. In our experimental settings of Trip13 depletion in HCC cells, DNA damage and apoptosis were highly induced during mitosis (Figure S2B, Supporting Information).

Our data indicated that Trip13 depletion in HCC cells induced cell death presumably as a consequence of prolonged and aberrant mitosis associated with elevated levels of DNA damage. To further explore the role of Trip13 in mitosis regulation, we decided to analyze the role of Aurora kinase A, an important regulator of centrosome and mitotic spindle formation.^[^ ^20^ ^]^ Immunofluorescence revealed a mislocalization of Aurora kinase A during mitosis (Figure S2C, Supporting Information), including the presence of multipolar foci indicative of multiple active MTOCs as observed before with pericentrin staining. The mislocalization of Aurora kinase A and its role in microtubule dynamics during mitosis prompted us to analyze the effect of Aurora kinase A KD in Trip13 KD cells. Remarkably, the KD of Aurora kinase A was sufficient to rescue proliferation in Trip13 KD cells (Figure 2I). Interestingly, Aurora kinase A depletion also counteracted elevated DNA damage levels and apoptosis (Figure 2J,K), and reduced mitotic index and normal appearance of mitotic cells in the Trip13 background (not shown). Of note, expression levels of Aurora kinase A in synchronized Trip13 KD cells was similar to controls, indicating that Aurora kinase A mislocalization, but not levels, was, at least in part, responsible for prolonged mitosis and induced DNA damage upon Trip13 depletion (Figure 2L).

Collectively, these data supported a model in which prolongation of mitosis, DNA damage, as well as mislocalization of spindle proteins were required components of Trip13‐KD‐dependent induction of apoptosis in HCC cells.

### 2.3 Trip13 KD Results in Lipid Droplet Accumulation through Activation of the Insulin Signaling Pathway

**Figure 3:**
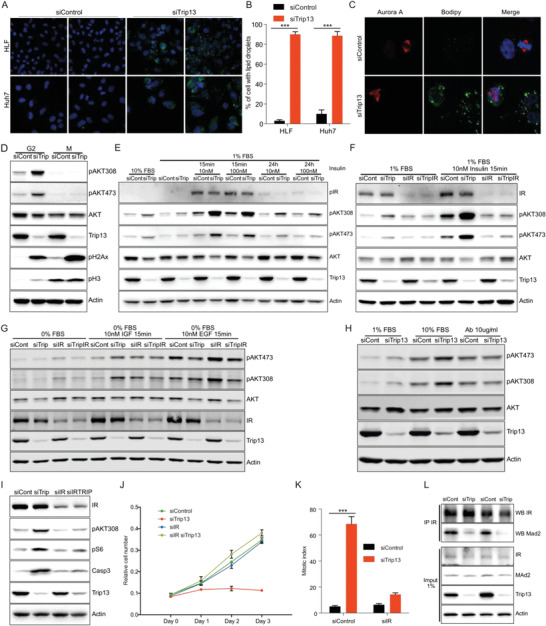
Trip13 KD results in lipid droplet accumulation through activation of the insulin signaling pathway. A) Representative images of HLF and Huh7 cells transfected with siControl or siTrip13 showing lipid droplet accumulation. Green (bodipy), blue (DAPI) (*n* = 3). B) Quantification of the proportion of HLF and Huh7 cells displaying lipid droplets under control and Trip13 KD conditions (*n* = 3). C) Immunofluorescence images of Trip13 KD HLF cells showing lipid droplets in interphase and in mitosis. Red (Aurora kinase A), blue (DAPI) (*n* = 3). D) Western blot analysis of Trip13 KD HLF cells synchronized in G2 or mitosis, showing levels of Akt phosphorylation. E) Levels of Akt phosphorylation in Trip13 KD HLF cells before and 15 min or 24 h after insulin treatment (10 or 100 nm) (*n* = 3). F) Western blot of HLF transfected with siTrip13 alone or in combination with siInsulin receptor (IR) showing levels of pAkt before and after 15 min treatment with insulin 10 nm (*n* = 3). G) Western blot of HLF transfected as in (F), showing levels of pAkt before and after 15 min treatment with 10 nm IGF or EGF subsequent 24 h total serum starvation (0% FBS) (*n* = 3). H) Levels of pAkt in Trip13 KD HLF cells before and 15 min after 10% serum stimulation in combination with 10 µg mL^−1^ of insulin receptor blocking antibody (*n* = 3). I) Western blot showing levels of pAkt and cleaved caspase 3, and J) growth curve of HLF cells with KD for Trip13 and/or insulin receptor (*n* = 3). K) Mitotic index of cells transfected as in (I) (*n* = 3). L) CoIP of insulin receptor and Mad2 in HLF cells transfected with siControl or siTrip13 (*n* = 3). All data in the figure are shown as the mean ± SEM. *n* numbers refer to biological replicates. (K) 1‐way ANOVA with Tukey's Multiple Comparison test. (B) Student's *t*‐test. *p < 0.05, **p < 0.01, ***p < 0.001.

As mitosis is a highly energy demanding process and Trip13 KD induced mitosis blockage prior to cell death, we hypothesized that metabolic adaptations were part of the Trip13 KD mitotic phenotype. Unexpectedly, we did not detect any changes in adenosine triphosphate (ATP), acetyl coenzyme A (Acetyl‐CoA), or nicotinamide adenine dinucleotide phosphate (NADPH) levels despite the prolonged mitosis observed in Trip13 KD cells (data not shown). Furthermore, Seahorse analysis of glucose, glutamine, and fatty acid oxidation did not show any significant differences between control and Trip13 KD cells (Figure S3A–C, Supporting Information). However, metabolic characterization including Bodipy staining for intracellular lipids revealed a marked accumulation of lipid droplets upon KD of Trip13 (**Figure** **3**A). Trip13‐KD‐dependent lipid droplet accumulation was consistently observed in the different HCC cell lines, increasing the proportion of lipid droplet detection from 5% to 10% in control to around 90% in Trip13‐depleted HLF and Huh7 cells (Figure 3A,B). Interestingly, lipid droplet accumulation was observed in interphase and mitotic HCC cells, indicating that lipid droplets did not reflect cell cycle exit or cell differentiation (Figure 3C).

Despite the fact that bodipy staining only detects neutral lipids, we wanted to evaluate if Trip13 KD also affected other lipid species. In line with the observed accumulation of lipid droplets, we observed a significant increase in triglyceride levels upon Trip13 KD with no changes in phospholipid or cholesterol levels (Figure S3D, Supporting Information). We next aimed to study the source of triglycerides in our cells. To this end, we determined the capacity of Trip13 KD cells to synthetize triglycerides from extracellular free fatty acids provided as oleate. As demonstrated before, Trip13 KD cells exhibited an increased triglyceride content when compared to control cells (Figure S3E, Supporting Information). However, in the presence of high extracellular oleate, we observed overall increased but not significantly different triglyceride (TG) contents in control and Trip13 KD cells (Figure S3E, Supporting Information). Therefore, increased free fatty acid (FFA) uptake could not explain the accumulation of lipid droplets and the increased TG levels observed in Trip13 KD cells, suggesting that de novo fatty acid synthesis and lipogenesis were the main sources for induced TG synthesis in our cells. To test this hypothesis, we measured TG content of Trip13 KD cells cultured in lipoprotein deficient serum or low glucose conditions. As expected, low glucose conditions significantly reduced TG content in Trip13 KD cells (Figure S3F, Supporting Information). By contrast, low lipoprotein containing media did not significantly reduce the TG content of the cells (Figure S3F, Supporting Information). Related to the decrease in TG content, low glucose conditions widely rescued cell growth in Trip13 KD cells, as well as attenuated the induction in apoptosis, DNA damage, and mitosis markers (Figure S3G,H, Supporting Information). These data suggest a relevant role of elevated TG storage in the induction of cell death mediated by Trip13 loss‐of‐function.

As insulin/Akt signaling is the major pathway in hepatic lipogenesis, we probed for Akt signaling upon Trip13 KD in HCC cells. To rule out the possibility of confounding differences in Akt activity due to the accumulation of mitotic cells upon Trip13 KD, we analyzed Akt phosphorylation in synchronized HLF cells in G2 and M phases. Interestingly, Trip13 KD was sufficient to markedly induce pAkt in G2 phase, whereas no activation was observed during mitosis (Figure 3D and Figure S3I (Supporting Information)).

These data uncovered a novel, mitosis‐independent function of Trip13 in the regulation of Akt signaling. Indeed, Trip13 KD increased pAkt levels, the effect of which was enhanced by insulin treatment depending on the concentration and the time point (Figure 3E). By contrast, genetic inhibition of insulin signaling by knocking down the insulin receptor (IR) prevented induced Akt phosphorylation by Trip13 KD in both, nonstimulated or insulin‐stimulated HCC cells (Figure 3F). Despite this, Trip13 KD cells starved in 1% fetal bovine serum (FBS) (used to not induce a strong stress response due to complete serum deprivation) still showed some degree of Akt activation when compared with control cells (Figure 3E,F). Of note, total serum starvation (0% FBS, i.e., complete deprivation of insulin and other growth factors) prevented Akt phosphorylation in response to Trip13 KD, demonstrating that Akt induction uponTrip13 loss‐of‐function was mediated by an extracellular signal (Figure 3G and Figure S3J (Supporting Information)). Furthermore, other growth factors such basic fibroblast growth factor (bFGF) or epidermal growth factor (EGF) did not induce Akt activation and even insulin‐like growth factor (IGF) showed only a minor activation Akt in response to Trip13 KD (Figure 3G and Figure S3J (Supporting Information)). Taken together, these data indicate that the observed induction of Akt phosphorylation (both pAkt308‐ and pAkt473‐dependent activation) upon KD of Trip13 was mediated by insulin signaling through the insulin receptor. To confirm this, we blocked the IR by using an IR antagonist peptide (S961) or a specific IR blocking antibody. In both approaches, the levels of Akt phosphorylation in Trip13 KD cells were restored to the levels of control cells (Figure 3H and Figure S3K (Supporting Information)). Importantly, the KD of the IR was able to rescue the effects of Trip13 KD on proliferation, apoptosis, and mitotic index (Figure 3I–K), demonstrating that activation of the IR pathway was an essential mechanistic component of the overall Trip13 KD phenotype. To corroborate these findings, we knocked down phosphatase and tensin homolog (PTEN), a phosphatase negatively regulating the Akt pathway, or overexpressed a constitutively active form of PI3K, a pathway component upstream of Akt. Activation of Akt signaling by these means increased proliferation of HCC cells (Figure S3L–O, Supporting Information), indicating that the previously observed cell death by Akt activation in HCC cells occurred specifically in the context of Trip13 KD. In agreement with the latter conclusion, no significant changes in mitotic index were observed in PTEN KD or phosphoinositide 3‐kinase (PI3K) overexpression (OE) cells (Figure S3P, Supporting Information).

These data revealed an as‐yet unknown and critical role of Trip13 in the regulation of the insulin/Akt signaling pathway and downstream lipid metabolism in HCC cells, suggesting that Trip13‐KD‐dependent Akt activation during interphase was required for the mitotic defects observed in HCC cells.

During mitosis, Trip13 is involved in the disassembly of the MCC by direct binding to p31^comet^,^[^ ^3^, ^13^, ^14^ ^]^ thereby inducing ATP‐hydrolysis‐dependent Mad2 inactivation by switching it to an open conformation, a process representing a prerequisite for proper chromosome segregation and mitotic exit.^[^ ^3^, ^21^ ^]^ Interestingly, the mitotic checkpoint components p31^comet^ and Mad2 have been shown to be also involved in the regulation of insulin receptor signaling, in which p31^comet^ is required for the inhibition of Mad2‐/Bubr1‐dependent IR endocytosis in hepatocytes.^[^ ^22^ ^]^ In the context of mitotic checkpoint regulation, Mad2 remains in the closed conformation in the absence of Trip13, which inhibits the binding to Bubr1 and prevents mitotic exit.^[^ ^3^ ^]^ Accordingly, we hypothesized that KD of Trip13 induces insulin signaling by preventing Mad2/Bubr1 binding and removal of the IR from the plasma membrane. Indeed, Mad2 KD in HCC cells also induced Akt phosphorylation and reduced cell proliferation, mimicking Trip13 KD effects (Figure S3Q,R, Supporting Information). We next aimed to confirm Mad2 binding to insulin receptor by co‐immunoprecipitation (CoIP). Consistent with our previous observations, Mad2 was found to bind to the IR and this interaction was markedly reduced upon Trip13 KD (Figure 3L).

Taken together, these data demonstrate for the first time that Trip13 regulated insulin signaling by inactivating Mad2 through the conversion from its closed to the open conformation, reminiscent of mechanisms described for MCC inhibition during mitosis.

### 2.4 Inhibition of Trip13‐KD‐Induced Accumulation of Lipid Droplets Rescues Mitotic Abnormalities and Cell Death

**Figure 4:**
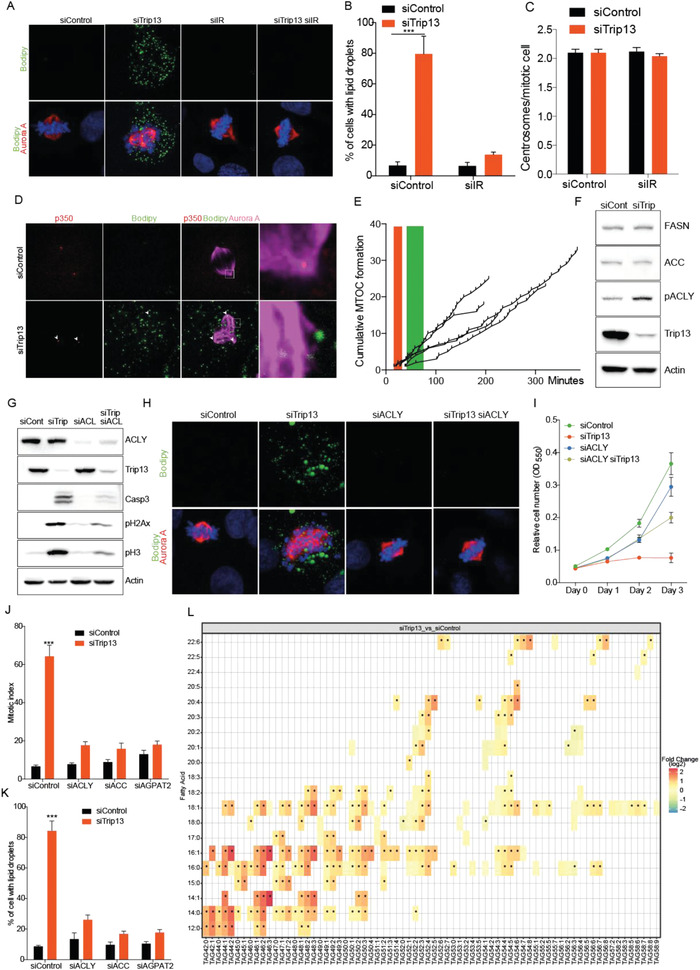
Prevention of Trip13‐KD‐induced accumulation of lipid droplets rescues mitotic abnormalities and cell death. A) Representative images of HLF control cells and cells upon KD of Trip13 and/or insulin receptor showing lipid droplets and mitotic spindles. Red (Aurora kinase A), green (bodipy), blue (DAPI) (*n* = 3). B) Quantification of the proportion of cells displaying lipid droplets transfected as in (A). C) Quantification of the number of centrosomes in mitotic HLF cells. D) Representative images of control and Trip13 KD HLF cells showing lipid droplets (Bodipy) and centrosomes (p350) (*n* = 3). E) Number of MTOCs formed during mitosis in Trip13 KD cells. The green bar represents the time of metaphase initiation in control cells, the red bar represents the time of anaphase in control cells (*n* = 5). F) Western blots showing FASN, ACC, and pACLY in Trip13 KD HLF cells (*n* = 4). G) Levels of cleaved caspase 3, pH2Ax, and pH3 in HLF control cells or upon KD of Trip13 and/or ACLY (*n* = 3). H) Representative images of HLF cells with KD of Trip13 alone or in combination with ACLY KD showing spindle abnormalities and lipid droplet accumulation (*n* = 3). I) Growth curve of HLF control cells or upon KD of Trip13 and/or ACLY (*n* = 3). J) Mitotic index of HLF cells upon Trip13 KD alone or in combination with KD of ACLY, ACC, or AGPAT2 (*n* = 3). K) Percentage of HLF cells displaying lipid droplets transfected as in (J) (*n* = 3). L) Lipidomic analysis showing log2 fold changes of TG concentrations between Trip13 KD (siTrip13) and control (siControl) HLF cells at 30 h post‐transfection (*n* = 5). The *x*‐axis represents triglycerides (TGs) with number of carbon atoms and number of double bonds. The *y*‐axis indicates one fatty acid (out of three per TG) that can be specified using the multiple reaction monitoring (MRM)‐based Lipidyzer assay. (B, C, J, K) Data are shown as the mean ± SEM and analyzed by 1‐way ANOVA with Tukey's Multiple Comparison test. *p < 0.05, **p < 0.01, ***p < 0.001. (L) Data were analyzed using unpaired Wilcoxon rank‐sum tests corrected for multiple comparisons using the Benjamini–Hochberg method. *q* values <0.05 (*) were considered as statistically significant. *n* numbers refer to biological replicates. See also Figure S4 in the Supporting Information.

Since Trip13 KD induced Akt activation during interphase resulted in lipid droplet accumulation, we decided to investigate whether the changes in de novo fatty acid and triglyceride synthesis pathway were involved in the observed dysregulation of mitosis in HCC cells. To this end, we first analyzed lipid droplets in mitotic cells in more depth. As shown before, Trip13 KD induced lipid droplet accumulation in mitotic cells. Interestingly, some lipid droplets were located at the edges of the multipolar spindles which formed upon Trip13 depletion (**Figure** **4**A). Strikingly, concomitant insulin receptor KD completely blunted Trip13‐KD‐dependent lipid droplet formation and restored bipolar spindles (Figure 4A,B), This is in line with the finding that IR KD reversed Trip13 KD effects on cell proliferation and mitotic index (Figure 3H,I). To gain further insights into the role of lipid droplets in spindle polarity, we determined the number of centrosomes during mitosis. Interestingly, based on p350 centrosomal antigen staining, Trip13 KD as well as control cells show two centrosomes per mitotic cell (Figure 4C,D). However, Trip13 KD showed multiple MTOCs arising from lipid droplets, the larger ones similar to centrosomal MTOCs and the smaller ones inducing spindle deformations (Figure 4D). Using live cell imaging in Trip13 KD HLF cells constitutively expressing H2B‐mCherry and Aurora kinase A–green fluorescent protein (‐GFP), aMTOCs were shown to be highly dynamic and abundant with a live time of a few minutes and cumulative number around 30 per mitosis (Figure 4E and Movies S1 and S2 (Supporting Information)). Interestingly, the first aMTOCs in Trip13 KD cells appeared at very early time points in mitosis corresponding with the time of prometaphase in control cells, and aMTOC accumulation continued until cell death. These data show for the first time that lipid droplets act as aMTOCs during mitosis, inducing aberrant microtubule nucleation, multipolar spindle formation, and eventually chromosome mislocalization and cell death.

To validate that lipid droplets were indeed responsible for mitotic aberrations, we next determined different components of the de novo lipid synthesis pathway in Trip13 KD cells. We did not find any increase in Acetyl‐CoA carboxylase (ACC) and fatty acid synthase (FASN) total protein expression in HCC cells upon KD of Trip13 (Figure 4F). However, we observed a marked increase in the activating phosphorylation of ATP‐citrate lyase (pACLY) (Figure 4F), representing a well‐established target of Akt catalyzing the synthesis of acetyl‐CoA required for de novo lipid synthesis. In order to exclude that lipid accumulation was a secondary consequence of reduced utilization due to mitotic arrest, we determined the levels of proteins involved in de novo lipid synthesis and markers of mitosis (pH3), DNA damage (pH2Ax), and cell death (Casp3), as well as intracellular TG levels at different time points after siRNA transfection (Figure S4A,B, Supporting Information). Importantly, Akt and ACLY phosphorylation upon Trip13 KD were induced already at 24 h post‐transfection, clearly before the induction of mitotic arrest, DNA damage, and cell death (Figure S4A, Supporting Information). However, minor or no increase was observed in FASN or ACC total protein levels at any time point (both normally induced by Akt), possibly due to negative selection of cells with high expression of these proteins. Furthermore, TG levels were already increased 24 h after transfection, demonstrating that lipid droplet accumulation preceded mitotic arrest and cell death upon Trip13 KD (Figure S4B, Supporting Information). The induction of pACLY upon Trip13 KD was consistent in human HCC cell lines, including the commonly used HepG2 cells (Figure S4C, Supporting Information), suggesting a role of induced lipid synthesis and particularly ACLY activation in the development of the Trip13‐KD‐induced phenotype. Indeed, ACLY KD was able to rescue the effect of Trip13 KD on cell proliferation, apoptosis, mitotic index, spindle polarity, and lipid droplet accumulation (Figure 4G–K). In order to rule out that the observed effects of ACLY KD were more related to its role in tumor cell aggressiveness by inducing acetyl‐CoA‐level‐dependent protein acetylation,^[^ ^23^ ^]^ we also performed the KD of ACC, the latter representing the enzyme catalyzing the following step in de novo lipogenesis utilizing acetyl‐CoA for fatty acid synthesis. Despite the fact that ACC was not upregulated under Trip13 KD, the combination of Trip13 and ACC KD in HLF cells also rescued the mitotic phenotype and increased cell proliferation by reducing lipid droplet accumulation (Figure 4J,K and Figure S4D–F (Supporting Information)).

Given that lipid droplets contain mainly TGs, we decided to further validate our findings on their role in Trip13‐KD‐dependent effects by depletion of acyl‐glycerol phosphate acyltransferase 2 (AGPAT2), one of the rate‐limiting enzymes in the TG synthesis pathway. Indeed, KD of AGPAT2 did not only reduce lipid droplet accumulation but also restored cell proliferation and prevented mitotic defects in Trip13 KD cells (Figure 4J,K and Figure S4D,G,H (Supporting Information)).

The data provided so far suggested that an induction of de novo lipogenesis (DNL) downstream of IR/Akt signaling was mainly responsible for TG accumulation contributing to mitotic aberrations upon Trip13 KD. In order to further exclude a relevant role of extracellular lipid uptake for the Trip13‐KD‐dependent phenotype, we knocked down CD36, an important membrane receptor involved in lipid uptake. In agreement with our previous data, CD36 KD did not rescue the effects of Trip13 KD on proliferation, apoptosis, DNA damage, mitotic arrest, or TG accumulation (Figure S4I,J, Supporting Information, and data not shown). Of note, the CD36 KD significantly reduced TG levels in Trip13 KD cells (Figure S4K, Supporting Information). However, this reduction was minor in comparison to the TG accumulation induced by Trip13 KD as well as to the rescue effects upon knock down of lipogenic genes (ACLY, ACC, AGPAT2), indicating that the de novo lipid synthesis was the main source of TGs accumulating upon Trip13 KD. To further validate these findings, we analyzed the TG composition in control and Trip13 KD HLF cells at the 30 h post‐transfection time point, in which TG levels are already increased but preceding massive induction of cell death, as previously demonstrated (Figure S4A,B, Supporting Information). As expected, the targeted lipidomic analysis showed that KD of Trip13 induced a marked elevation in TG levels when compared with control cells (Figure 4L). Interestingly, the strongest fold changes were observed for TGs containing between 42 and 48 carbon atoms, i.e., TGs consisting of combinations of C12, C14, and C16 saturated and monounsaturated fatty acids (Figure 4L). While C12, C14, and – to the largest extent – C16 fatty acids can be directly synthesized with the fatty acid synthase or after shortening of C16,^[^ ^24^ ^]^ their monounsaturated counterparts need an additional dehydrogenation step via desaturases.^[^ ^25^ ^]^ Altogether, these compounds can be considered as the primary products of de novo fatty acid synthesis. Most interestingly and representing surrogate markers for de novo lipid synthesis, we observed strong increases in TGs containing monounsaturated fatty acids (C14:1, C16:1, C18:1), indicating a higher activity of desaturases in response to Trip13‐KD‐induced DNL (Figure 4L). This is further supported by higher product‐to‐precursor ratios of the Δ9‐desaturase (D9D.C16:1/C16:0; D9D.C18:1/C18:0) in siTrip13 KD cells (Figure S4L,M, Supporting Information). In line with our previous findings, the results from the lipidomics measurements confirmed an increase in DNL as a result of the Trip13 KD. Collectively, our data demonstrated that lipid droplet accumulation through IR‐/Akt‐driven DNL, rather than acetyl‐CoA pool size or exogenous lipid uptake, was critical for the mitotic aberrations observed upon Trip13 KD, eventually resulting in HCC cell death.

### 2.5 The Lipid Droplet Coat Protein Perilipin‐2 Is a Key Component of the Trip13‐KD‐Induced Mitotic Phenotype

**Figure 5:**
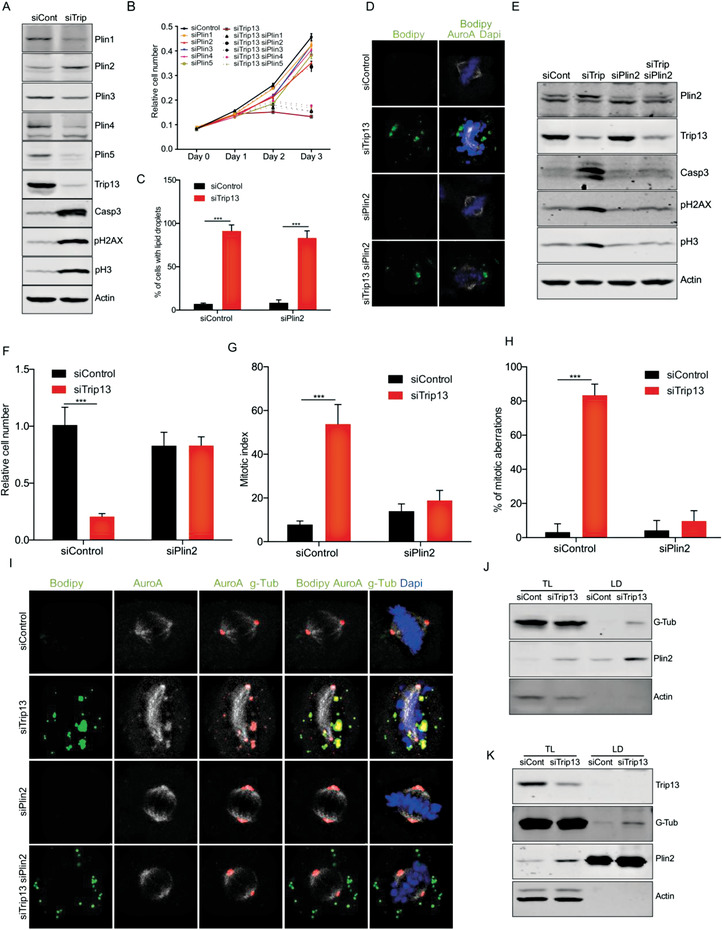
The lipid droplet coat protein perilipin‐2 is a key component of the Trip13‐KD‐induced mitotic phenotype. A) Western blot showing levels of perilipins 1–5 in Trip13 KD HLF cells (*n* = 3). B) Growth curve of HLF cells transfected with siTrip13 alone or in combination with siPlin1–5 (*n* = 3). C) Quantification of lipid droplets accumulation in cells transfected with siTrip13 alone or in combination with siPlin2 (*n* = 4). D) Representative images of HLF cells transfected as in (C) (*n* = 3). E) Western blot of HLF cells transfected with siTrip13 alone or in combination with siPlin2, showing levels of apoptotic, DNA damage and mitotic markers (*n* = 4). F) Relative cell number, G) mitotic index, and H) mitotic aberrations in HLF cells KD for Trip13 alone or in combination with Plin2 KD. I) Representative images of HLF cells KD for Trip13 alone or in combination with Plin2 KD, showing localization of Aurora kinasa A, g‐tubulin, and lipid droplets (*n* = 3). J) Western blot of total lysate or lipid droplets fraction from HLF cells transfected with siTrip13 (*n* = 3). K) Western blot of total lysate or lipid droplets fraction from HLF cells transfected with siTrip13 and treated with 200 µm oleate (*n* = 2). All data are shown as the mean ± SEM. *n* numbers refer to biological replicates. (C, F, G, H) 1‐way ANOVA with Tukey's Multiple Comparison test. *p < 0.05, **p < 0.01, ***p < 0.001. See also Figure S5 in the Supporting Information.

Our data suggested that lipid droplets contribute to Trip13‐KD‐induced cell death by serving as aMTOCs inducing multipolar spindle formation. To gain further insights into the underlying mechanisms, we investigated whether lipid droplet coat proteins were involved in the development of the mitosis phenotype. First, we determined the levels of the five perilipins in HCC cells upon KD of Trip13. Despite the fact that all five perilipins were expressed in HLF cells, perilipin‐2 was the only one showing increased levels upon Trip13 KD (**Figure** **5**A and Figure S5A (Supporting Information)). To determine if any of these lipid droplet coat proteins was involved in the mitotic phenotype, we knocked down the five perilipins in combination with Trip13 KD. Concomitant perilipin‐1 KD did not rescue the Trip13 KD effects (Figure 5B and Figure S5B (Supporting Information)). Instead, KDs of perilipin‐2, ‐4, and ‐5 were able to reduce the levels of apoptosis, DNA damage, and mitosis blockage induced by Trip13 KD (Figure S5B, Supporting Information). Notably, KD of perilipin‐2 showed the strongest rescue effect on apoptosis, DNA damage, and mitosis blockage when compared with the other perilipins, and was the only interference with a perilipin protein family member able to restore cell proliferation (Figure 5B and Figure S5B (Supporting Information)).

It has been described that binding to lipid droplets stabilizes perilipin‐2, and this binding protects lipid droplets from degradation.^[^ ^26^ ^]^ Also, it was previously described that perilipin‐2 regulates the insulin/Akt signaling pathway.^[^ ^27^ ^]^ As we had shown that the reduction in lipid droplets by KD of the IR, ACLY, ACC, and AGPAT2 rescued the Trip13‐KD‐mediated mitotic phenotype, we were wondering whether the rescue induced by perilipin‐2 was also associated with a reduction in lipid droplet accumulation or insulin signaling attenuation. Strikingly, the KD of perilipin‐2 rescued Trip13‐KD‐mediated effects on proliferation, DNA damage, and cell death, but exerted only minor effects on lipid droplet formation (Figure 5C–F). Moreover, Plin2 KD in HCC cells did not affect insulin signaling in control or Trip13 KD cells (Figure S5C, Supporting Information). This suggested that lipid droplets coated with perilipin‐2, and not lipid droplets per se, were essential for mediating mitotic cell death induced by Trip13 KD. Given that our previous data indicated that the formation of lipid droplets was required for the mitotic aberrations and mitotic arrest in Trip13 KD cells, we decided to further investigate whether the lipid droplets observed in perilipin‐2 KD cells still retained these functions. Interestingly, the mitotic index of Trip13 KD cells was restored to control levels by perilipin‐2 KD (Figure 5G). Moreover, mitotic aberrations and aMTOC amplification were completely rescued by perilipin‐2 KD (Figure 5H). To validate these findings, we also studied the role of perilipin‐2 in other HCC cell lines. As expected, Trip13 KD in HepG2 cells increased protein levels of perilipin‐2 (Figure S5D, Supporting Information). Also, the KD of perilipin‐2 in HepG2 cells rescued the Trip13 KD phenotype, without affecting Akt regulation, thereby confirming the effects observed in HLF cells (Figure S5D, Supporting Information).

As perilipin‐2‐coated lipid droplets were essential for Trip13 KD effects, we were wondering if loading of HCC cells with lipids would be sufficient to affect mitosis progression. HLF cells treated with 200 µm oleate did not shown any changes in mitotic, DNA damage, or apoptotic markers (Figure S5E, Supporting Information). Also, IF of these cells showed larger lipid droplets than the ones observed upon Trip13 KD. Interestingly, KD of Trip13 in cells treated with oleate still induced the mitotic defects described above, which were rescued by Plin2 KD (Figure S5F, Supporting Information). As oleate treatment had no effect on Plin2 levels in HLF cells, we were wondering if palmitate, a more toxic fatty acid, could affect Plin2 levels. Treatment of HLF cells with 200 µm palmitate increased Plin2 levels and induced the apoptotic marker Casp3, but (in contrast to Trip13 KD) without affecting mitosis progression (Figure S5G, Supporting Information). Next, we speculated that Trip13 regulated the amount of Plin2 binding to lipid droplets. To test this hypothesis, we decided to study the levels of Plin2 protein in lipid droplets of control and Trip13 KD cells treated with oleate. WB of purified lipid droplets from those cells did not show any evident changes in the amounts of Plin2 (Figure S5H, Supporting Information).

To understand how lipid droplets could affect mitosis under Trip13 KD conditions, we performed IF for proteins involved in spindle pole organization. Encouragingly, Trip13 KD induced the colocalization of lipid droplets with g‐tubulin and Aurora kinase A, key components of microtubules organization (Figure 5I). Furthermore, Plin2 KD restored the normal spindle localization of g‐tubulin and Aurora kinase A in the context of Trip13 KD (Figure 5I). Also, western blot analysis of purified lipid droplets confirmed that Trip13 KD induced g‐tubulin interaction with these organelles, not only in basal conditions, but also in cells loaded with oleate (Figure 5J,K).

In summary, these data demonstrated that metabolic alterations induced by Trip13 KD resulted in the formation of perilipin‐2‐coated lipid droplets. This perilipin‐2 component of the accumulating lipid droplets was essential for the recruitment of microtubule organization components and aMTOC generation, leading to mitotic aberrations and cell death.

### 2.6 Perilipin‐2 Confers Susceptibility to Trip13‐KD‐Induced Cell Death

The data provided above demonstrated that Trip13 KD induced a complex phenotype in HCC cells, involving changes in lipid metabolism associated with mitotic aberrations and arrest as well as DNA damage. Importantly, all these changes were required for the resulting induction of cell death. To determine whether this newly discovered mechanism also extended to other tumor entities beyond HCC, we determined the effects of Trip13 KD in different tumor cell lines. In order to gain specific insights from these experiments, we selected two cell lines that, in accordance with the human protein atlas, express Trip13.

**Figure 6:**
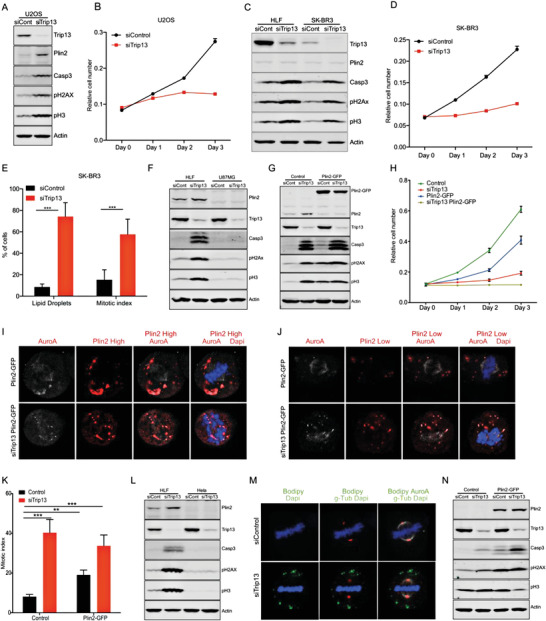
Perilipin‐2 confers susceptibility to Trip13‐KD‐induced cell death. A) Western blots showing levels of cleaved caspase3 pH2Ax and pH3 in Trip13 KD U2OS cells (*n* = 4). B) Growth curve of U2OS cells transfected as in (A). C) Western blot of HLF and SK‐BR3 cells transfected with siControl or siTrip13 showing levels of cleaved caspase3 pH2Ax and pH3 (*n* = 3). D) Growth curve of Trip13 KD SK‐BR3 cells. E) Lipid droplet accumulation and mitotic index of SK‐BR3 cells KD for Trip13. F) Western blot of HLF and U87MG cells transfected with siTrip13 showing levels of cleaved caspase3 pH2Ax and pH3 (*n* = 3). G) Western blot of HLF wt or Plin2 overexpressing cells transfected with siTrip13, showing levels of cleaved caspase3 pH2Ax and pH3 (*n* = 3). H) Growth curve of cells transfected as in (G). I) Representative images of HLF Trip13 KD cells with high or J) low levels of Plin2 overexpression showing Plin2 and Aurora kinase A localization (*n* = 3). K) Mitotic index of cells transfected as in (G) (*n* = 3). L) Western blot of HLF and HeLa cells transfected with siTrip13 showing levels of Plin2, cleaved caspase3, pH2Ax, and pH3 (*n* = 2). M) Representative images of HeLa cells KD for Trip13 showing lipid droplets (*n* = 3). N) Western blot of HeLa wt or Plin2‐overexpressing cells transfected with siTrip13, showing levels of cleaved caspase3 pH2Ax and pH3 (*n* = 3). All data are shown as the mean ± SEM. *n* numbers refer to biological replicates. (E, K) 1‐way ANOVA with Tukey's Multiple Comparison test. *p < 0.05, **p < 0.01, ***p < 0.001. See also Figure S6 in the Supporting Information.

We first studied the effect of Trip13 KD in the osteosarcoma cell line U2OS. Western blot analysis of Trip13 KD in U2OS showed increased levels of perilipin‐2 and elevated markers of mitotic arrest, DNA damage, and apoptosis, associated with a marked reduction in cell growth (**Figure** **6**A,B). In accordance with our previous results in HCC cells, Trip13 KD also increased lipid droplet accumulation, mitotic index, and mitotic aberrations (Figure S6A–C, Supporting Information). To confirm that the mitotic phenotype observed in U2OS upon Trip13 KD was also dependent on the corresponding metabolic dysregulation, we knocked down ACC and perilipin‐2 in combination with Trip13 KD. ACC KD partly rescued Trip13 KD effects (Figure S6D–F, Supporting Information), whereas the KD of perilipin‐2 completely rescued the Trip13 KD phenotype (Figure S6D–F, Supporting Information). Interestingly, the rescue induced by perilipin‐2 KD was independent of lipid droplet accumulation, confirming our HCC data that perilipin‐2‐coated lipid droplets were specifically responsible for the mitotic arrest and cell death induced by lowering Trip13 levels.

Next, we also studied the effect of Trip13 KD in SK‐BR3 human breast cancer cells. This cell line showed rather low levels of Trip13 under basal conditions when compared with HCC cells (Figure 6C). Nonetheless, knocking down Trip13 in these cells was sufficient to induce markers of mitotic arrest, DNA damage, and apoptosis, all of them to comparable levels as previously observed in HCC cells (Figure 6C). In congruence with our previous results in HCC cells, the reduction in cell growth upon KD of Trip13 in SK‐BR3 was associated with increased lipid droplet accumulation and mitotic arrest (Figure 6D,E), suggesting that induction of cell death by Trip13 KD was not necessarily determined by its basal levels in different tumor entities. Therefore, assessing relative Trip13 expression levels will not be sufficient to predict susceptibility or resistance of tumors to its inhibition.

Since our results showed that perilipin‐2‐coated lipid droplets were required for the aMTOC formation and induction of cell death upon Trip13 KD, we hypothesized that low levels of perilipin‐2 conferred resistance to Trip13 KD effects. To test this hypothesis, we focused on the glioblastoma cell line U87MG, which based on publicly available data (https://www.ebi.ac.uk/gxa/home), is characterized by very low expression levels of perilipin‐2. Indeed, Plin2 protein levels were undetectable in U87MG when compared with HLF cells even upon Trip13 KD (Figure 6F). Interestingly, Trip13 KD in U87MG cells did not exert significant mitotic arrest, DNA damage, or expression of apoptotic markers (Figure 6F). Despite the fact that U87MG cells carry an inactivating mutation of PTEN that results in a high Akt phosphorylation, Trip13 KD in these cells increased Akt activity still further, also reflected by the induction of ACLY phosphorylation (Figure S6G, Supporting Information). Of note, also FASN and ACC protein levels were increased upon Trip13 KD in U87MG. These findings supported our hypothesis in such that we did not observe induced FASN and ACC protein levels in the cell lines undergoing cell death upon Trip13 KD, such as HLF cells. This was potentially due to a strong negative selection of cells exhibiting a strong lipogenic response. Thus, U87MG cells lacking Plin2 still showed Akt activation and elevated levels of proteins involved in de novo lipogenesis, but were resistant to Trip13‐induced mitotic arrest and cell death. This suggested that depletion of Trip13 induced mitotic cell death only in presence of Plin2. To test this assumption, we generated a lentivirus vector used for constitutive expression of a Plin2–GFP fusion protein in HLF cells. Strikingly, overexpressing Plin2 alone in this cell line was sufficient to increase the levels of mitotic, DNA damage, and apoptotic markers (Figure 6G). Moreover, IF of Plin2–GFP alone or in combination with Trip13 KD colocalized with Aurora kinase A in cells with low or high overexpression levels (Figure 6I,J), and Plin2–GFP OE reduced proliferation associated with a significant increase in mitotic index (Figure 6H,K). While all the effects of Plin2 overexpression mimicked the Trip13 KD phenotype to some extent, we did not detect any changes in Trip13 levels. The combination of Trip13 KD with Plin2 overexpression did not further increase the effects of Trip13 KD alone in terms of mitosis phenotype, DNA damage, and apoptosis (Figure 6G–K). However, we were able to detect an increase in the percentage of nuclear abnormalities in interphase cells, indicative of mitotic failure, when Plin2 overexpression was combined with Trip13 KD (Figure S6H,I, Supporting Information). Finally, we overexpressed perilipin‐2–GFP fusion protein in U87MG, a cell line without any detectable expression of perilipin‐2, as shown in Figure 6F. The overexpression of perilipin‐2 alone in U87MG cells, either upon transfection or lentivirus transduction, had no detectable effects on the measured parameters (Figure S6J–M, Supporting Information). However, in agreement with our HCC cell line data, the combination of Trip13 KD with perilipin‐2 overexpression resulted in a pronounced increase in markers of mitotic arrest, DNA damage, and apoptosis, which was reflected in a dramatic reduction of cell growth (Figure S6J–M, Supporting Information).

As most descriptions of Trip13 regulation of mitosis were done in HeLa cells, we decided to check if our findings would be also applicable to this extensively investigated cell line. Due to resistance to Trip13 KD previously described for HeLa cells, we hypothesized that Plin2 expression levels in this cell line should be very low. Indeed, KD of Trip13 in HeLa cells did not induce any markers of defective mitosis, DNA damage, or apoptosis (Figure S6N, Supporting Information). Moreover, and in agreement with our hypothesis, HeLa cells did not display any detectable levels of Plin2 when compared with HLF cells (Figure 6L). Of note, Trip13 KD in HeLa cells did not induce mitotic abnormalities, despite Akt activation and lipid droplet accumulation observed in cells in mitosis and interphase (Figure 6M and Figure S6O,P (Supporting Information)). Intriguingly, Plin2 overexpression in HeLa cells induced high levels of DNA damage and apoptosis markers, and these effects were stronger than the ones induced by Trip13 KD alone, overall without major changes in the mitotic marker pH3 (Figure 6N). The combination of Trip13 KD with Plin2 overexpression induced a further increase in DNA damage and apoptotic markers, associated with a marked reduction in cell number (Figure 6N and Figure S6Q (Supporting Information)). Due to the fact that the HeLa cells seemed not to be blocked in mitosis upon Trip13 KD, in contrast to other cell models, we decided to investigate if Plin2 overexpression was involved in mitotic aberrations that could explain the observed DNA damage and cell death. Interestingly, mitotic index was drastically reduced in this cell line when Trip13 KD was combined with Plin2 overexpression, in accordance with the pH3 levels (Figure S6R, Supporting Information). To further investigate if Plin2 overexpression in HeLa cells was only inducing a mitotic exit in the context of Trip13 KD or was also affecting mitosis fidelity, we analyzed the nucleus of cells in interphase. Plin2 overexpression was enough to cause a significant increase in micronucleus containing cells and binucleated cells, indicating a failure during mitosis (Figure S6R–T, Supporting Information). Furthermore, when Plin2 overexpression was combined with Trip13 KD, we observed a drastic increase in micro‐, bi‐, and multinucleated cells, explaining the increase in DNA damage and reduced cell number observed under these conditions (Figure S6R–T, Supporting Information).

Together, our results revealed a new role of Trip13 in the regulation of lipogenesis contributing to Trip13‐KD‐induced cell death of tumor cells from different cancer entities, showing for the first time that Plin2 was indispensable for mitotic cell death induction under conditions of SAC disturbance.

### 2.7 High Perilipin‐2 Levels Sensitize Tumor Cells to Mitosis‐Targeting Paclitaxel

Our findings provided mechanistic insights relevant for the exploitation of future Trip13 inhibitors for cancer therapy by defining Plin2 as predictive marker for the susceptibility of tumors to a respective targeted therapy in the context of a precision medicine approach.

**Figure 7:**
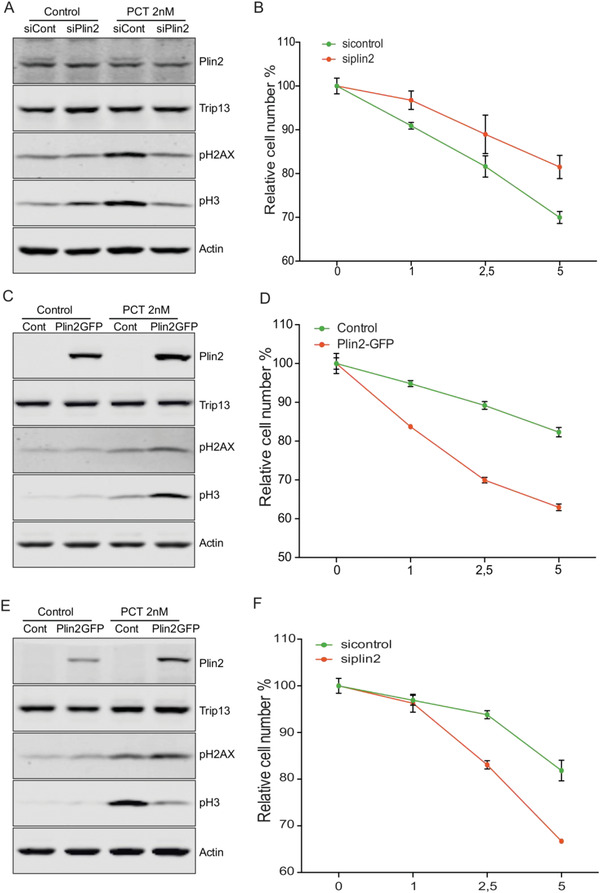
High perilipin‐2 levels sensitize tumor cells to mitosis‐targeting paclitaxel. A) Western blot of HLF cells transfected with siPlin2 and treated with 2 nm paclitaxel for 48 h, showing levels of pH2Ax and pH3 (*n* = 3). B) Growth curve of HLF cells transfected as in (A) and treated with 0–5 nm paclitaxel (*n* = 3). C) Western blot of U87MG control or Plin2 overexpressing cells treated with 2 nm paclitaxel for 48 h, showing levels of pH2Ax and pH3 (*n* = 3). D) Growth curve of U87MG cells transfected as in (C) and treated with 0–5 nm paclitaxel (*n* = 3). E) Western blot of HeLa wt or Plin2‐overexpressing cells treated with 2 nm paclitaxel for 48 h, showing levels of pH2Ax and pH3 (*n* = 3). F) Growth curve of HeLa cells transfected as in (E) and treated with 0–5 nm paclitaxel (*n* = 3). All data are shown as the mean ± SEM. *n* numbers refer to biological replicates.

Despite the fact that a Trip13‐specific inhibitor (DCZ0415) has been recently described, showing a potent antitumor activity in multiple myeloma in vitro and in vivo,^[^ ^19^ ^]^ it is not expected to be in the clinics any time soon. Consequently, we were wondering if Plin2 was affecting cell survival when treated with clinically approved mitosis‐targeting drugs. To this end, we focused on paclitaxel, a microtubule poisoning agent acting on the cells by a microtubule stabilizing mechanism. Remarkably, paclitaxel treatment (1–5 nm) in HLF cells with KD of Plin2 displayed lower levels of DNA damage and mitotic markers when compared with control cells, which translated to an increased resistance of paclitaxel‐induced cell death (**Figure** **7**A,B). Furthermore, U87MG cells overexpressing Plin2 and treated with paclitaxel showed higher levels of DNA damage and mitotic markers, with reduced cell survival compared to control cells (Figure 7C,D).

Finally, we also studied if Plin2 expression could affect paclitaxel sensitivity of HeLa cells. Interestingly, and as we expected, Plin2 overexpression induced higher levels of DNA damage and reduced survival in combination with reduced mitotic markers, when compared to control cells (Figure 7E,F). In summary, our results demonstrated that Plin2 was required for induction of mitotic aberrations and cell death in different tumor entities, implying that Plin2 could be a predicting marker of susceptibility to future tumor treatments affecting SAC and to microtubule‐poisoning drugs already in clinical use.

## 3 Discussion

Mitosis is an essential biological process and its dysregulation represents one of the defining features of tumor cells. As such, mitosis has been used as a common target in different human cancers from almost the beginning of chemotherapy. Despite the fact that the mechanism of action of respective chemicals is based on the activation of the SAC leading to a prolonged mitotic arrest and eventually cell death, these drugs are only effective in a subset of cancer patients. However, the tumor characteristics that define and predict the therapeutic response to these drugs are still mainly unknown.

In this context, we show that the SAC regulating protein Trip13 is commonly increased in mouse and human HCC as compared to normal livers, which is in agreement with previous reports from various cancer entities.^[^ ^16^ ^]^ Moreover, we demonstrate that Trip13 depletion in proliferating HCC cells strongly interferes with mitosis, causing aberrations in spindle formation, mitotic arrest, and eventually mitotic cell death. Previous publications demonstrated that Trip13 loss‐of‐function reduces tumor growth in head and neck cancer and colon cancer, as well as more recently in HCC.^[^ ^16^ ^]^ The oncogenic effects of Trip13 have mainly been attributed to an enhanced capacity for DNA damage repair, particularly through DNA–dependent protein kinase c (DNA–PKc)‐dependent nonhomologous end joining, thereby contributing to genomic instability.^[^ ^16^ ^]^ In fact, we were able to prevent Trip13‐KD‐induced cell death by interfering with the DNA damage response through inactivation of the ATM/ATR pathway. Notably, while we observed a modest increase in DNA damage immediately after Trip13 KD, the gradually increasing portion of HCC cells displaying mitotic arrest suggests that DNA damage accumulates during prolonged mitosis contributing a combinatorial mechanism resulting in mitotic cell death upon diminished Trip13 levels.

As outlined in the introduction, various studies contributed to the characterization of the role of Trip13 in cell cycle control, defining its function in p31^comet^‐dependent MCC disassembly and mitotic exit,^[^ ^13^, ^14^, ^28^ ^]^ or proposing a dual role in SAC activation and inactivation.^[^ ^29^ ^]^ While there might be cell‐type‐dependent differences, Trip13's regulatory function in mitosis has been attributed to the capacity of this ATPase to remodel the mitotic checkpoint protein Mad2, affecting Mad2 complex forming properties.^[^ ^21^, ^30^ ^]^ The mitotic arrest that we observed upon Trip13 depletion in HCC cells is in line with its function as a MCC silencing protein enabling mitotic exit. However, the present study reveals a new role of Trip13 in the regulation of lipid metabolism in liver cancer cells. Specifically, mitotic cell death upon Trip13 depletion in HCC cells was dependent on insulin receptor/Akt signaling, which resulted in cellular lipid droplet accumulation acting as functional aMTOCs. Of note, these findings widely exclude that the observed mitotic arrest upon Trip13 KD could be explained by the previously described inhibition of APC/C due to mitotic DNA damage, the mechanism of which is not completely elucidated.^[^ ^31^ ^]^

While the role of Trip13 in mitotic checkpoint complex regulation has been studied extensively, not much is known about other functions related to its ATPase activity, the latter of which was shown to catalyze conformational changes in substrate proteins involved in regulatory processes.^[^ ^21^ ^]^ In this respect, it is of particular interest that a recent publication proposed a role of the MCC proteins p31^comet^ and Mad2 in metabolic control.^[^ ^22^ ^]^ The authors showed in hepatocytes that Mad2 binds to the insulin receptor and is involved in endocytosis of the receptor upon insulin binding. IR endocytosis is inhibited by p31^comet^ binding to Mad2, stabilizing unstimulated IR. However, beyond showing the p31^comet−/−^ displayed glucose intolerance and insulin resistance, the study has not further elucidated the relevance of this mechanism under specific physiological or pathophysiological conditions, particularly not a role in IR‐/Akt‐signaling‐dependent tumor cell death. As mentioned above, Trip13 is required for Mad2 binding to MIM containing proteins. Indeed, our findings now demonstrate that Trip13 regulates Akt activation, mainly mediated through the IR (a MIM containing protein), with Trip13 being a key player in the p31^comet^‐/Mad2‐dependent regulation of insulin signaling in liver cancer cells.

Interestingly, very recent studies revealed a functional link between intracellular lipids and MTOC formation in cancer associated fibroblasts,^[^ ^32^ ^]^ as well as in prostate tumor cells.^[^ ^33^ ^]^ In proliferating prostate cancer cells, inhibition of the lipogenesis gene diacylglycerol o‐acyltransferase 1 (DGAT1) exerted antiproliferative effects due to dual regulatory functions on lipid storage as well as on aMTOC protein levels, suggesting a role of aMTOCs in tumor growth and aggressiveness.^[^ ^33^ ^]^ We now provide data supporting a different mechanism in which accumulating Plin2‐coated lipid droplets acts as functional aMTOCs which induce aberrant mitotic spindle formation and mitotic failure in cancer cells. Strikingly, we could demonstrate that the lipid droplets acting as aMTOCs accumulate as the result of increased insulin/Akt signaling and de novo lipogenesis upon Trip13 KD, defining a novel combinatorial mechanism of induced mitotic cell death. Different studies have shown that the metabolic status of oocytes (a model of aMTOCs) is very important for the fertility and the survival of these cells.^[^ ^34^ ^]^ Notably, increased accumulation of lipid droplets and misdistribution in oocytes of mice under high‐fat diet (HFD) feeding conditions correlates with an increase in inappropriate progression of meiosis mainly due to spindle defects detected in more than 50% of the oocytes studied.^[^ ^35^ ^]^ While the mechanistic role of lipid droplets in oocyte function remains widely unclear, the spindle defects observed in oocytes of HFD‐fed animals resemble very much the same aberrations that we described in Trip13 KD cells, suggesting that lipid droplets could also act as aMTOCs interfering with spindle orientation in meiosis.

Given the lack of a more specific definition, as long as cell death processes are activated within the duration of mitosis leading to cell death during mitosis or thereafter, this is regarded as mitotic cell death or mitotic catastrophe.^[^ ^36^ ^]^ Different anticancer drugs target the highly regulated and therefore vulnerable process of mitosis, including those interfering with microtubule dynamics or regulatory kinases, such as inhibitors of Chk1, as well as novel ones holding the promise of improving cancer treatment in the future.^[^ ^7^, ^37^ ^]^ Interestingly, mitotic cell death or mitotic catastrophe is known to be induced by DNA damage in mitosis, and multipolar spindles are characteristic of this process.^[^ ^36^ ^]^ Here, we show that lipid droplet accumulation, concretely perilipin‐2‐coated lipid droplets, is required for multipolar spindle formation and cell death in the context Trip13 KD or antimitotic drugs such as paclitaxel. Thus, it would be interesting to investigate whether perilipin‐2 levels in different human tumors influence the efficiency of therapeutic inducers of mitotic catastrophe to activate this cell death pathway. It is also tempting to speculate that the combination of DNA‐damaging agents with inducers of lipid accumulation, such us cyclic adnosine monophosphate (cAMP) mimics or novel inhibitors of Trip13 could exert synergistic antitumor effects.

Lipid droplets had already been shown to be involved in the mitosis process in *Drosophila* taking on a dual role of energy supply and protein buffering. Concretely, *Drosophila* lipid droplets bind various proteins, but histones are the most extensively studied. Moreover, binding of histones to lipid droplets seems to be mediated by Jabba on the surface of lipid droplets.^[^ ^38^, ^39^ ^]^ Due to this, it is tempting to speculate that Trip13/Plin2 could have a similar role in mammals as Jabba does in *Drosophila*, by regulating the binding of mitotic proteins to lipid droplets and thus inducing aMTOC formation.

## 4 Conclusion

Our research demonstrates a new role of lipid droplets in mitosis, but how perilipin‐2 is able to “transform” lipid droplets into functional aMTOCs remains an open question that is beyond the objective of this paper and will need further investigation. However, the described functional link of Trip13‐lipid metabolism and especially Plin2 in the regulation of mitosis, provides novel implications for cell biology as well as for the development of antimitotic treatment strategies against cancer.

## 5 Experimental Section

### Mouse Experiments

Male mice were obtained from Charles River Laboratories (Brussels, Belgium) and maintained on a 12 h light–dark cycle at room temperature with regular unrestricted diet and free access to water. For inducing HCC in mice, C3H/HeNCrl mice (Charles River) were injected with 25 mg kg^−1^ DEN or vehicle at 2 weeks of age. Mice were killed at 26 and 30 weeks and tumors were dissected from the liver and snap‐frozen for further analyses. For subcutaneous tumor growth experiments, Hepa1‐6, Huh7, or HLF cells were transduced with lentiviruses described below expressing shControl or shTrip13 and selected with puromycine for 3 days. 2 × 10^6^ cells were injected into the left flank of BALB/c nude mice (CAnN.Cg‐Foxn1nu/Crl, Charles River) and tumor growth was monitored twice a week for two to four weeks depending on the implanted cell line and tumor growth, after which mice were sacrificed for tissue sampling. Animal handling and experimentation was performed in accordance with the European Union directives and the German animal welfare act (Tierschutzgesetz) and approved by local authorities (Regierungspräsidium Karlsruhe; approval number 35‐9185.81/G‐177/11).

### Cell Culture, Growth Curves, Mitotic Index, and Apoptosis

HLF, Hepa1‐6, Huh7, HepG2, U2OS, SK‐BR3, U87MG, and HeLa cell lines were maintained in Dulbecco's modified Eagle's medium (DMEM) (Gibco) supplemented with 10% FBS (Millipore), and cultured at 37 °C, 5% CO_2_, and 95% humidity. Growth curves were monitored using the MTT Cell Proliferation Kit (Roche Diagnostics GmbH, Germany) or crystal violet staining. Cell proliferation was assessed using the BrdU Cell Proliferation Assay Kit (New England Biolabs). Insulin, IGF, EGF, or bFGF stimulation was performed in cells cultured in DMEM supplemented with 0–1% FBS for 12–24 h and treated with 10–100 nm insulin (Sigma I9278), 10 nm IGF‐I (PeproTech 100‐11), 10 nm EGF (PeproTech AF‐100‐15), 10 nm bFGF (PeproTech 100‐18B), or 10% FBS for 15 min or 10–100 nm insulin for 24 h (as indicated in the figure legends). Insulin receptor blocking experiments were performed in cells cultured in DMEM supplemented with 1% FBS for 24 h and treated with 10 µg mL^−1^ insulin blocking antibody (Fisher Scientific, InVivoMAb anti‐human CD220 (Insulin Receptor), Clone: IR 83‐22, Ref.: 50‐206‐5623) or 0.1–1 µm insulin receptor peptide antagonist (NovoNordisk, S961) for 30 min prior to the 10% FBS stimulation lasting 15 min. Cell synchronization was obtained by double thymidine/RO‐3306 treatment as described elsewhere.^[^ ^40^ ^]^ Briefly, cells were treated with 2 mm thymidine (Sigma 89270) for 17 h, released from the blocking growth media without thymidine for 9 h, and then subjected to a second thymidine block for 14 h. During the second thymidine block, cells were transfected with siControl or siTrip13, released for 2 h, and treated for 8 h in growth media containing RO‐3306 (10 µm) (Sigma SML0569). Cells in G2 were harvested immediately after release from the RO‐3306 block and mitotic cells were selected by mitotic shake‐off 1 h after RO‐3306 release. For assessing growth curves, 12 h after transfection, cells were seeded in 24‐well plates and fixed every 24 h. Relative cell number was determined by measuring absorbance at OD550 of crystal violet stained cells. For determination of the mitotic index, cells were seeded 12 h after transfection in 24‐well plates, and stained at different time points with Hoechst‐33258. Total cell number and mitotic cells in each condition were counted in 5 independent fields of each triplicate and represented as percentage of mitotic cells. Apoptosis was quantified by Apo‐ONE Caspase3/7 assay from Promega (G7790) according to the manufacturer's instructions.

### Metabolic Flux and Lipid Levels

Mitochondrial respiration and glycolytic flux were measured with the Seahorse Bioscience XF Extracellular Flux Analyzer (Agilent Technologies). After transfection, cells were seeded in XF cell culture plates (Agilent Technologies 102416‐100) and cultured for 24 h before medium replacement with Seahorse XF DMEM (Agilent Technologies 103575‐100) supplemented with 1 mm pyruvate, 2 mm l‐glutamine for glycolytic rate assay or 1 mm pyruvate, 2 mm l‐glutamine, and 10 mm glucose for Mito stress and Mito fuel flex assays. The experiments were performed following manufacturer's instructions. Concretely, after basal measurements, compounds were added sequentially to the cells as follows. Glycolytic rate assay (Agilent Technologies 103344‐100), 10 mm glucose, 0.5 µm rotenone, and 50 mm 2‐deoxyglucose. Cell Mito stress assay (Agilent Technologies 103015‐100), 1.5 µm oligomycin, 1 µM carbonyl cyanide‐ptrifluoromethoxyphenylhydrazone (FCCP), and 0.5 µm rotenone/actimycin. For Mito fuel flex assay (Agilent Technologies 103260‐100), 2 µm UK5099, 3 µm bi‐2‐(5‐phenyacetamido‐1,2,3‐thiadiazol‐2‐yl) ethyl sulfide (BPTES), or 4 µm Etomoxir were used in different combinations following manufacturer instructions. After normalization to the protein content, data were analyzed and represented by using the Agilent Technology software.

Triglycerides, cholesterol, or phospholipids were quantified by triglyceride determination kit (Spinreact 1001310), cholesterol determination kit (Spinreact 100010193), or phospholipids assay kit (Abcam ab234050), following manufacturer's instructions. In detail, cells were resuspended in phosphate‐buffered saline (PBS) at different time points after transfection, as indicated in the corresponding figures. Protein concentration was determined by bicinchoninic acid (BCA) assay and lipids were extracted in 2:1 chloroform:methanol. After normalization of the volumes by protein concentration, lipid assays were performed and data represented as percentage of the controls.

### Lipidomic Analysis

1 × 10^6^ HLF cells were seeded in 6‐well plates and transfected with siControl or siTrip13 as mentioned above. For each sample, 30 h post‐transfection, cells from 2 wells of a 6‐well plate were recovered in dry ice cooled 80% methanol (1 mL) and immediately stored at −80° until analysis was performed. Five siControl and five siTrip13 cell samples were homogenized in 80% MeOH with 320 mg glass beads (0.5 mm, VK‐05, PeqLab) using a PeqLab Precellys24 homogenizer. Samples were cooled to 0–3 °C and homogenized twice at 5500 rpm for 25 with 5 s breaks in‐between. The homogenate was further used for three purposes. First, 250 µL of the homogenate was extracted with methyl‐*tert*‐butyl ether according to the protocol published by Ghorasaini et al.^[^ ^41^ ^]^ Second, 100 µL of each sample homogenate was pooled to a total volume of 100 µL and extracted in triplicates in the same way as the individual study samples for quality control purposes. Finally, the cell count was estimated using DNA levels based on fluorescence labeling with the Hoechst dye (final concentration: 20 µg mL^−1^ in PBS) as described by Muschet et al.^[^ ^42^ ^]^ Lipids were quantified using the Sciex Lipidyzer platform as described in Morigny et al.^[^ ^43^ ^]^

All lipidomic data processing and analyses steps were conducted using the R Statistical language (version 4.1.0; R Core Team, 2021). Concentration values were normalized to cell numbers using the results from the Hoechst assay. A three‐step procedure was used to ensure high data quality: first, lipids containing more than 35% of missing values in the pool samples were discarded from the data set (*n* = 480). Furthermore, lipids having more than 50% of missing values in each study group (siTrip13, siControl) were discarded (*n* = 8). In a second step, lipids with a coefficient of variation >25% in the pool samples were removed from the data set (*n* = 11). The last quality control step comprised the calculation of the dispersion ratio (*D*‐ratio) for each lipid.^[^ ^44^ ^]^ All in all, 173 lipids had a *D*‐ratio larger than 50%, indicating that the variability of lipid species concentrations in the biological study samples was predominantly based on technical variance. After the quality control process, 398 lipid species from 13 lipid classes remained in the data set. Remaining missing values (*n* = 140, equivalent to 3.5% of the total data points) were imputed using the KNN‐obs‐sel imputation method by Do et al.^[^ ^45^ ^]^ with *K* = 5 nearest neighbors. The activities of desaturases were estimated by calculating product‐to‐precursor ratios as described in Gerl et al.^[^ ^46^ ^]^

### Antibodies

The following Cell Signaling Technology antibodies were used: ATM (13934), perilipin‐1 (#9349), ACC (#3662), ACLY (#13390), pACLY (#4331), carbohydrate response element binding ptotein (ChREBP) (#58069), carnitine palmitoyl transferase 1 (CPT1) (#12252), stearyl‐coenzyme A desaturase (SCD1) (#2794), Akt (#4691 or #9272), pAktS473 (#9271), pAktT308 (#13038), phospho‐mammalian target of rapamycin (pmTOR) (#5536), pS6 (#2215), pp70S6K (#9234), pH2Ax (#9718), pH3 (53348), cyclin B1 (#12231), phospho‐insulin receptor (pINSR) (#3026), cleaved caspase‐3 (#9661), actin (#5125), PTEN (#9188). Plin1 (ab61682), Plin2 (ab108323 and ab52356), Plin3 (ab47638), Plin4 (ab234752), Plin5 (ab222811), sterol regulatory‐element binding protein (SREBP) (ab28481), Mad2 (ab97777), GFP (ab290), pericentrin (ab4448), CD36 (ab133625), tubulin (ab7291), and valosin‐containing protein (VCP) (ab11433) were ordered from abcam, P350 (ATA‐AMAB91164‐100) from Biozol, Trip13 (19602‐1‐AP) from Proteintech, and INSR (sc‐711), FASN (sc‐20140) from SantaCruz Biotech.

### Plasmids, Viral Constructs, and RNA Interference

pLKO.1 containing scrambled control shRNA was obtained from Addgene (#1864), pLKO.1 vector sets with shRNA against TRIP13 were obtained from Sigma‐Aldrich. The following clones resulted in strong knockdown of Trip13 and were selected for further experiments: clones TRCN0000022063 and TRCN0000022059 for human TRIP13, TRCN0000319690 for mouse TRIP13. pLenti6‐H2B‐mCherry was a gift from Torsten Wittmann (Addgene plasmid # 89766). pHR_dSV40‐Aurora A–GFP was a gift from Ron Vale (Addgene plasmid # 67924). pEGFP‐C1‐ADRP was a gift from Elina Ikonen (Addgene plasmid # 87161). pLenti6‐ADRP was generated by subcloning. pBabe puro HA PIK3CA E545K was a gift from Jean Zhao (Addgene plasmid # 12525). psPAX2 (Addgene plasmid # 12260) and pMD2.G (Addgene plasmid # 12259), both were a gift from Didier Trono.

ON‐TARGETplus siRNAs were from Dharmacon: siControl (D‐001810‐01), siTrip13 (L‐016262‐00), siAurora A (L‐003545‐01), siACLY (L‐004915‐00), siACC (L‐004551‐00), siAGPAT2 (L‐003811‐00), siCD36 (L‐010206‐00), siINSR (L‐003014‐00), Mad2 (L‐003271‐00), siPten (L‐003023‐00), siMad2 (L‐003271‐00), siPlin1 (L‐019595‐01), siPlin2 (L‐019204‐01), siPlin3 (L‐015979‐00), siPlin4 (L‐184319‐00), siPlin5 (L‐033568‐01).

### Transfection and Infection

For KD experiments using siRNAs, the different cell lines were transfected in 6‐well plates with Lipofectamine RNAiMax combined with 10 nm of the different ON‐TARGETplus siRNAs from Dharmacon as indicated. 12 h after transfection, cells were trypsinized and seeded for the corresponding assays. For siRNA/DNA cotransfection, cell lines were seeded in 6‐well plates and transfected with Lipofectamine 2000 combined with 10 nm of the ON‐TARGETplus siRNAs from Dharmacon and 1 µg of plasmid DNA. For lentivirus generation, 1 × 106 HEK293T cells were seeded in 6‐well plates in DMEM 10% FBS the day before transfection. 1 µg of pLenti6‐H2B‐mCherry, pHR_dSV40‐Aurora A–GFP, pLenti6‐ADRP, or pLKO vectors were transfected in the cells with Lipofectamine 2000 (Invitrogen 11668027), in combination with 1 µg psPAX2 and 100 ng pMD2.G. 48 h later, the supernatant was recovered, filtered, and used to infect the cells.

### Quantitative Taqman Reverse Transcriptase‐Polymerase Chain Reaction

Total RNA was extracted from pulverized, homogenized tissue or cultured cells using Qiazol reagent (QIAGEN, Hilden, Germany) and RNeasy (Qiagen, Hilden, Germany). cDNA was prepared by reverse transcription using the M‐MuLV enzyme and Oligo dT primer (St. Leon‐Rot, Germany). cDNA was amplified using assay‐on‐demand kits, the ABIPRISM 7700 Sequence detector (Darmstadt, Germany) and the StepOne Plus software (Darmstadt, Germany). RNA expression data were normalized to levels of TATA‐box binding protein RNA.

### Western Blot Analyses and Immunoprecipitation

Cells were lysed at 4 °C in 300 µL of IP lyses buffer (Pierce 87787) containing protease (Roche 11697498001) and phosphatase (Roche 04906837001) inhibitors, and sonicated. Cell extracts were centrifuged 14 000× RPM for 5 min 4 °C and the supernatant collected to protein concentration determination with BCA protein assay (Pierce 23225). 20 µg of total protein was subjected to SDS‐PAGE electrophoresis, and after transfer, membranes were blocked and incubated overnight at 4 °C with primary antibodies. After secondary antibody incubation for 1 h at room temperature, signals were visualized by Chemidoc Image System (BioRad) and ImageLab software or Odyssey image system (Li‐COR) and ImageStudioLite software. For co‐immunoprecipitation of the insulin receptor, 400 µg protein lysate was precleared with 20 µL of protein A/G agarose beads (SantaCruz biotech sc‐2003). After agarose beads were removed (14 000× RPM 4 °C), supernatants were incubated with 5 µg of the insulin receptor antibody or GFP antibody overnight at 4 °C under rotation. On the following day, 20 µL of protein G agarose beads was added to the samples and incubated for 1 h at 4 °C under rotation. After washing the immune complex 5 times with lysis buffer, proteins were eluted in glycine pH3.5 and run by western blot. For co‐immunoprecipitation of Mad2, Pierce Co‐IP kit (ThermoFisher Scientific 26149) was used following the manufacturer`s instructions.

### Western Blot Quantification

Densitometric quantifications from Chemidoc Image System (BioRad) acquired images were analyzed with ImageJ software and from ImageLab software or Odyssey image system (Li‐COR) acquired images were analyzed with ImageStudioLite software. Quantifications of the different proteins were normalized by total protein for phospho‐Akt, by SREBP‐P for SREBP‐M or by actin for all other proteins from 3 independent experiments and represented as percentage of the control in each experimental setting. The quantification of all Western blots included in the paper are provided in Figures S7–S10 (Supporting Information) and the Supporting Information figure legends refer to specific Western blots in the paper.

### Immunofluorescence

Immunofluorescence was performed in cells grown on glass slides (Thermo Fisher FALC354108). After fixation with 4% paraformaldehyde, cells were permeabilized by incubation with 0.5% Triton X‐100 in PBS, 5 min. Blocking was performed with 3% bovine serum albumin, 0.2% Triton X‐100, 0.02% Tween‐20 in PBS for 1 h, and the slides were incubated with primary antibodies (Tubulin, perilipin, Aurora kinase A, p350) at 4 °C overnight. Slides were incubated with secondary antibodies, donkey anti‐rabbit IgG Alexa Fluor 488 or 555 (Thermo Fisher A‐21206 and A‐31572) and donkey anti‐mouse Alexa Fluor 488 or 555 (Thermo Fisher A‐21202 and A‐31570) for 1 h at room temperature. Lipids were stained with Bodipy (Life Technologies D3922), which was added with the secondary antibodies. All the washing steps were performed at room temperature for 10 min in 0.2% Triton X‐100, 0.02% Tween‐20 in PBS. The slides were mounted with ProLong Diamond Antifade Mountant with 4´,6‐diamino‐2‐phenylindole (DAPI) (Life Technologies P36962), and pictures were taken using a Laser Scanning Confocal Microscope (Olympus Fluoview 1200, Olympus, Tokyo, Japan) equipped with an Olympus UPlanSApo 60× 1.35 and an UPlanSApo 40× 1.25 Sil Oil immersion objective (Olympus, 37 Tokyo, Japan).

### Live Cell Imaging

Stable cells expressing H2B‐mCherry alone or in combination with Aurora A–GFP were seeded in 8‐well µ‐Slides (Ibidi 80826), 12 h after transfection with siControl or siTrip13 as described above. Live imaging was performed on a Leica microscope equipped with a humidity (95%), temperature (37 °C), and CO_2_ (5%)‐controlled chamber (Leica TCS SP5 Confocal, Leica Microsystems). Cell pictures were taken every minute and used to quantify time from nuclear envelope breakdown (NEBD) to anaphase or MTOC dynamics. Movies were generated with ImageJ software.

### Human Data

Trip13 mRNA expression levels in human liver samples shown in Figure 1A were determined using publically available data (E‐GEOD‐25097; https://www.ebi.ac.uk/arrayexpress). Gene expression analysis of Trip13 in human nontumor and HCC tissue samples (Figure 1C) was performed in a HCC cohort (GSE14520) from the Liver Cancer Institute and Zhongshan Hospital, Fudan University which was published previously (GSE14520).^[^ ^47^ ^]^ For western blot analysis (Figure 1D and Figure S1B (Supporting Information)), 19 liver samples from patients with HCC (17 male/2 female; mean age 70.9 ± 1.3 years) from tumor (T; *n* = 19) and nontumorous tissue (NT; *n* = 19) were obtained from patients undergoing surgical resection for HCC. Matched T and NT tissues were derived from the same patient. Diagnosis of HCC was made by computed tomography and by magnetic resonance imaging in selected cases. Serological markers of hepatitis B virus and hepatitis C virus were assessed for all patients, as well as anti‐human‐immunodeficiency‐virus, which was negative for all cases. All patients were treated surgically and received the same postoperative care by the same team of surgeons in the Charité‐Universitätsmedizin Berlin. Diagnosis was confirmed by a histologist and the surrounding tumor free tissue was also evaluated by the histologist. The study was approved by the local ethics committee (Ethikkommission der Charité‐Universitätsmedizin Berlin, Germany, Approval Number EA4/080/14), and patients gave their written informed consent to participate in the study. HCC was confirmed and graded as poorly (*n* = 4), moderately, (*n* = 10), or well differentiated (*n* = 4) or multifocal (*n* = 1). Tumor‐surrounding liver tissue samples from the same patients were confirmed as nontumorous by the histopathologist.

### Statistical Analysis

All data were presented as mean ± standard error of the mean (SEM). Normal variance test was performed before subsequence analysis. Comparisons were analyzed using Student's *t*‐test, one‐or two‐way Analysis of variance (ANOVA) using GraphPad Prism (GraphPad Software, San Diego). Differences were considered statistically significant at *p* < 0.05. See individual figure legends for specific statistical details.

## Conflict of Interest

The authors declare no conflict of interest.

## Supporting information

Supporting Information

Supplemental Movie 1

Supplemental Movie 2

## Data Availability

The data that support the findings of this study are available from the corresponding author upon reasonable request.
