# Supplementary material for: Trip13 Depletion in Liver Cancer Induces a Lipogenic Response Contributing to Plin2‐Dependent Mitotic Cell Death
Source: Adv Sci (Weinh). 2022 Aug 28;9(29):2104291. doi: 10.1002/advs.202104291 (PMC9561781; doi:10.1002/advs.202104291)
Supplement: Supplementary file 1 — Supporting Information [file ADVS-9-2104291-s002.pdf]

ADVANCED SCIENCE
Open Access

Supporting Information

for *Adv. Sci.*, DOI 10.1002/advs.202104291

Trip13 Depletion in Liver Cancer Induces a Lipogenic Response Contributing to Plin2-Dependent Mitotic Cell Death

Marcos Rios Garcia, Bettina Meissburger, Jessica Chan, Roldan M. de Guia, Frits Mattijssen, Stephanie Roessler, Andreas L. Birkenfeld, Nathanael Raschzok, Fabien Riols, Janina Tokarz, Maude Giroud, Manuel Gil Lozano, Goetz Hartleben, Peter Nawroth, Mark Haid, Miguel López, Stephan Herzig* and Mauricio Berriel Diaz**

Supporting Information

Trip13 depletion in liver cancer induces a lipogenic response contributing to Plin2-dependent mitotic cell death

Marcos Rios Garcia, Bettina Meissburger, Jessica Chan, Roldan M. de Guia, Frits Mattijssen, Stephanie Roessler, Andreas L. Birkenfeld, Nathanael Raschzok, Fabien Riols, Janina Tokarz, Maude Giroud, Manuel Gil Lozano, Goetz Hartleben, Peter Nawroth, Mark Haid, Miguel López, Stephan Herzig*, Mauricio Berriel Diaz**

Figure S1

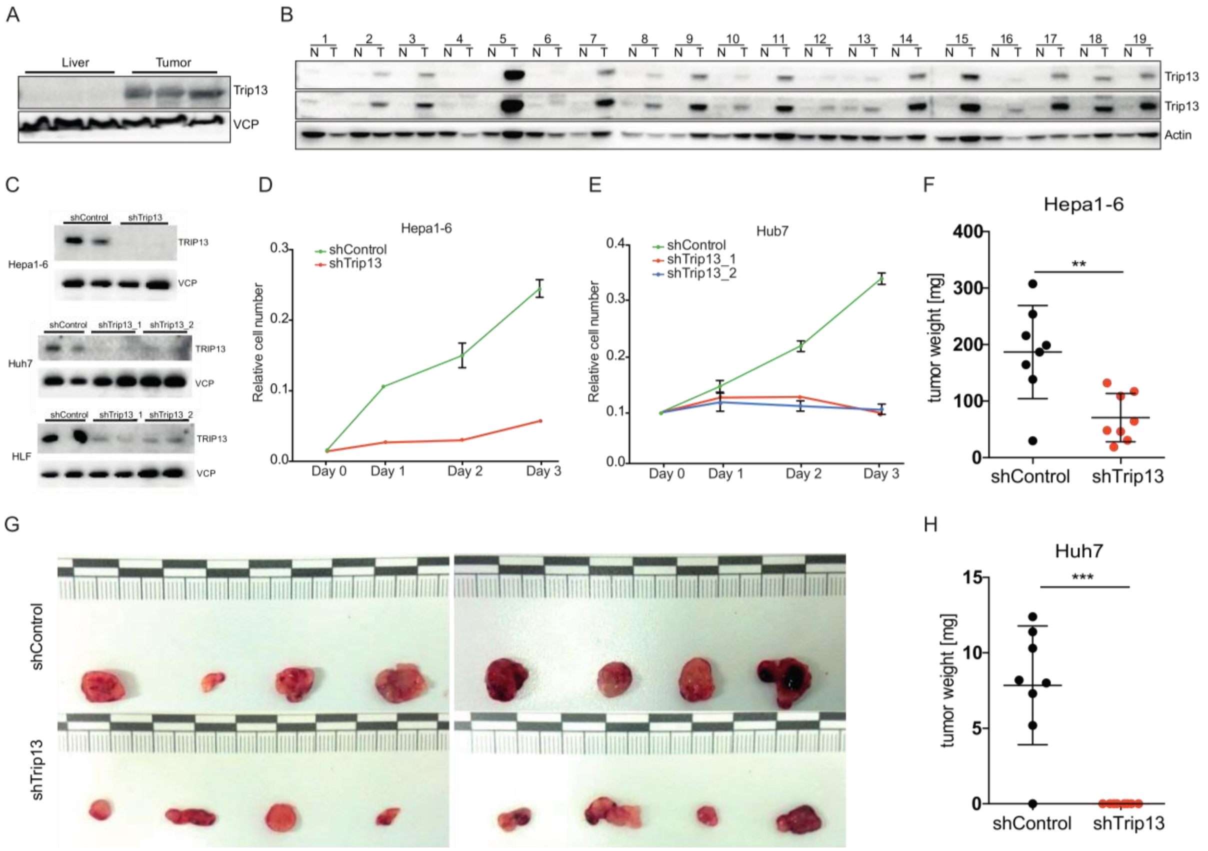

Figure S1. Trip13 is induced in human and mouse hepatocellular carcinoma. A) Western blot showing Trip13 levels in normal mouse liver or liver tumors induced by dimethylnitrosamine (DEN) treatment. B) Western blot of Trip13 levels in non-malignant human liver tissue, N, or matched hepatocellular carcinoma, T. (n=19). C) Western blot analysis of Hepa1-6, Huh7 and HLF cells showing KD levels of Trip13. D) Growth curve of Hepa1-6 and E) Huh7 cells infected with control shRNA or two different Trip13-targeting shRNAs. F) Tumor weight and G) macroscopic images of tumors from Hepa1-6 shControl or shTrip13 injected mice. H) Tumor weight of Huh7 shControl or shTrip13 cells. All data are shown as the mean ± s.e.m. n numbers refer to biological replicates. (F, H) Student's t-test. *P < 0.05, **P < 0.01, ***P < 0.001.

Figure S2

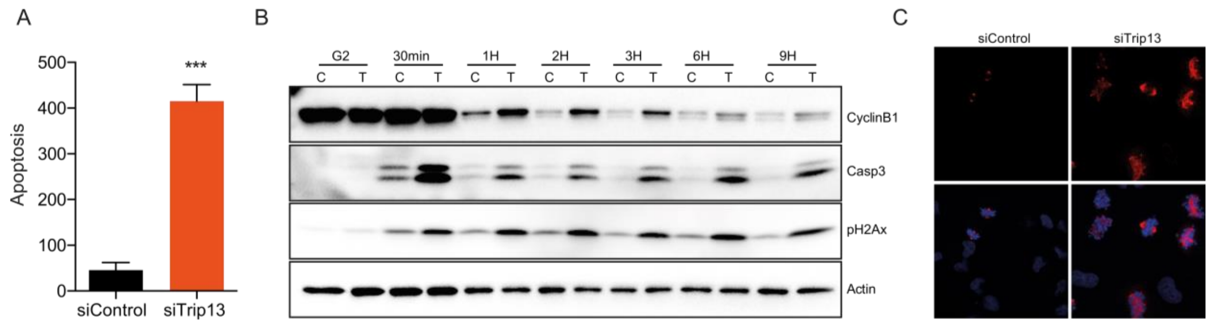

Figure S2. Loss of Trip13 triggers mitotic failure to induce cell death. A) Apoptosis levels in Trip13 KD HLF cells. B) Western blot analysis of Cyclin B1 cleaved capsase3 and pH2Ax in G2 synchronized and released HLF cells transfected with siControl “C” or siTrip13 “T”. C) Representative immunofluorescence images of siControl or siTrip13 transfected HLF cells. Red (Aurora kinase A), blue (dapi). All data are shown as the mean ± s.e.m. n numbers refer to biological replicates. A) Student’s t-test. *P < 0.05, **P < 0.01, ***P < 0.001.

Figure S3

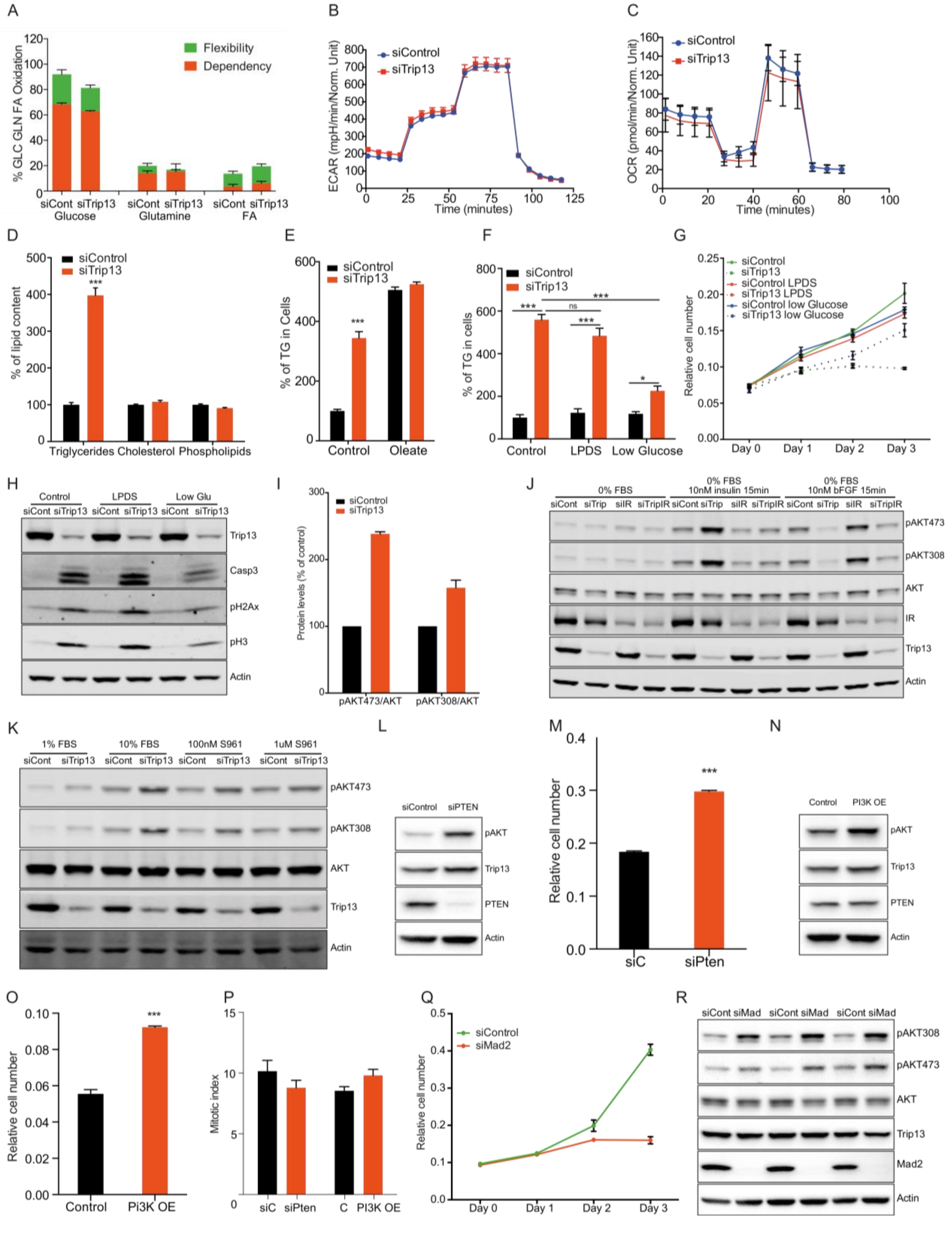

Figure S3. Trip13 KD results in lipid droplet accumulation through activation of the insulin signaling pathway. A) Oxygen consumption ratio in HLF control or Trip13 KD cells 30h post transfection showing glucose, glutamine or fatty acids dependency and flexibility. B) Glycolytic flux measured as extracellular acidification ratio of control or Trip13 KD cells 30h post transfection. C) Mitochondrial stress test showing oxygen consumption ratio in cells like in B. D) Levels of triglycerides, cholesterol and phospholipids in control and Trip13 KD cells.

E) Levels of triglycerides in 200uM oleate treated HLF control or Trip13 KD cells. F) Levels of triglycerides in HLF control or Trip13 KD cells growth in control, lipoprotein deficient or low glucose medium. G) Growth curve of cells treated like in F. H) Western blot analysis of cells as in F, showing levels of apoptosis, DNA damage and mitosis markers. I) Densitometry quantification of western blots showing levels of pAKT473 or pAKT308 normalized by total AKT levels in control and Trip13 KD cells. J) Western blot of HLF transfected with siTrip13 alone or in combination with siInsulin receptor (IR) showing levels of pAKT before and after 15min treatment with 10nM insulin or bFGF after 24h total serum starvation (0%FBS). K) Levels of pAKT in Trip13 KD HLF cells before and 15min after 10% serum stimulation in combination with different concentrations of insulin receptor blocking peptide (S961). L) Western blot analysis of PTEN KD HLF cells showing levels of AKT phosphorylation. M) Relative cell number of HLF control cells or upon KD for PTEN at day 3 after seeding. N) Western blot analysis of HLF cells overexpressing a constitutively active PI3K showing AKT phosphorylation levels. O) Relative cell number of cells from C at day 3 after seeding. P) Mitotic index of HLF control cells or cells with PTEN KD or overexpressing a constitutively active PI3K. Q) Western blot and R) growth curve of HLF cells transfected with siControl or siMad2. All data are shown as the mean ± s.e.m. n numbers refer to biological replicates. (E, F) 1-way ANOVA with Tukey's Multiple Comparison Posttest. (A, D, I, M, O, P) Student's t-test. $^{*}P < 0.05$, $^{**}P < 0.01$, $^{***}P < 0.001$.

Figure S4

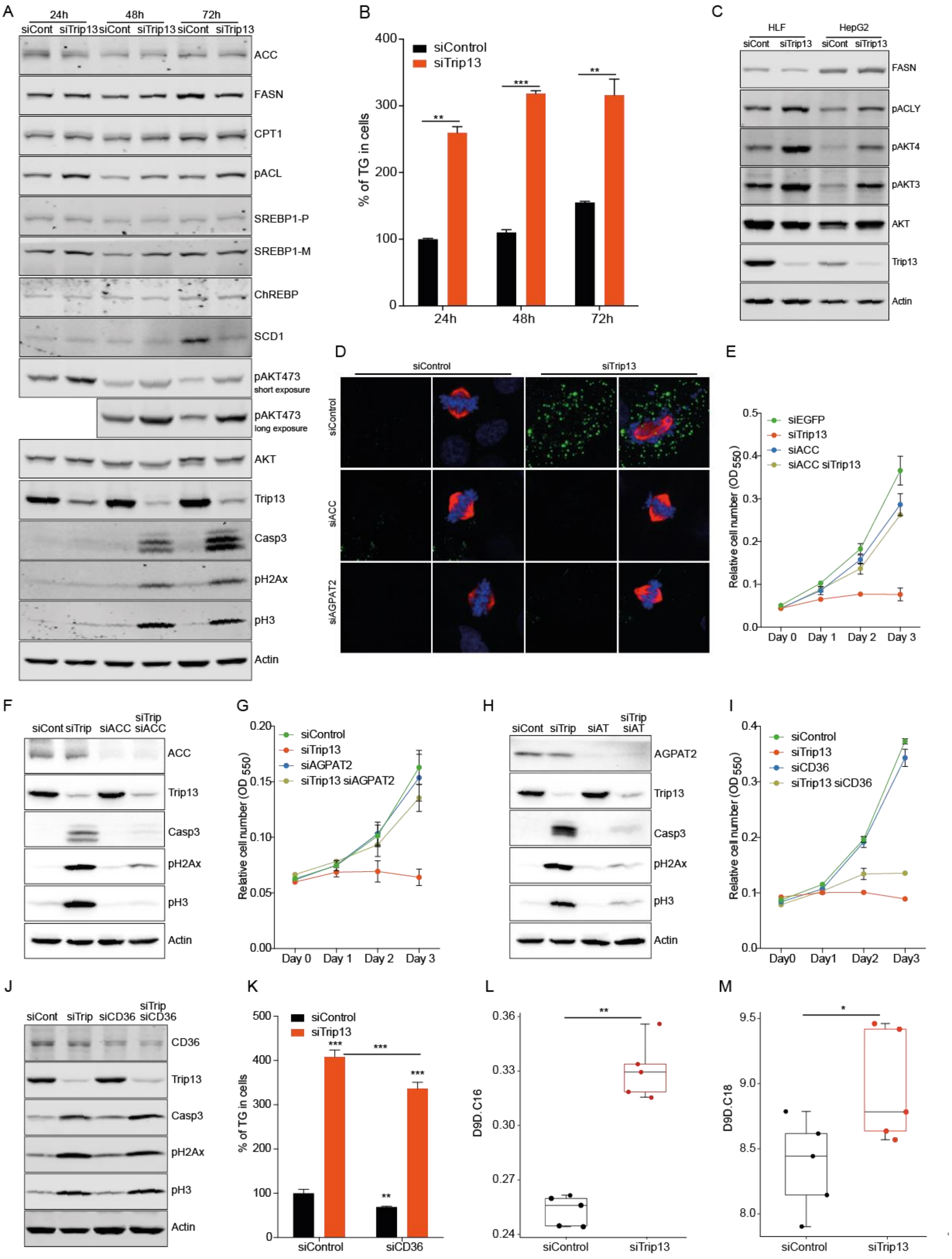

Figure S4. Prevention of Trip13 KD-induced accumulation of lipid droplets rescues mitotic abnormalities and cell death. A) Western blot analysis of HLF control or Trip13 KD cells showing levels of AKT activation, lipogenic proteins, apoptosis, DNA damage and mitosis markers 24h 48h or 72h post transfection. B) Triglyceride content of cells from A. C) Western blot of HLF or HepG2 cells showing AKT activation, FASN and pACLY levels after Trip13

KD. D) Representative images of HLF control cells and upon KD for Trip13 alone or in combination with ACC or AGPAT2 KD showing lipid droplets and spindle morphology. Green (Bodipy), Red (Aurora kinase A), blue (dapi). E) Growth curve of HLF control cells or upon Trip13 and/or ACC KD. F) Western blot analysis of cells from A showing apoptosis, DNA damage and mitotic markers. G) Growth curve and H) Western blot analysis of HLF control cells and upon Trip13 and/or AGPAT2 KD. I) Growth curve and J) Western blot analysis of HLF control cells and upon Trip13 and/or CD36 KD. K) Triglyceride content of cells from J. L) Product-to-precursor ratios used as proxies to estimate the enzyme activity of of the Δ9-desaturase D9D.C16 (Σ C16:1/C16:0) or M) D9D.C18 (Σ C18:1/C18:0) in Trip13KD (siTrip13) and control (siControl) cells at 30h post-transfection. (B, K) Data are shown as the mean ± s.e.m. 1-way ANOVA with Tukey's Multiple Comparison Posttest. (L, M) Data were analysed using unpaired Wilcoxon rank-sum tests. P-values <0.05 were considered as statistically significant. n numbers refer to biological replicates. $^{*}P < 0.05$, $^{**}P < 0.01$, $^{***}P < 0.001$.

Figure S5

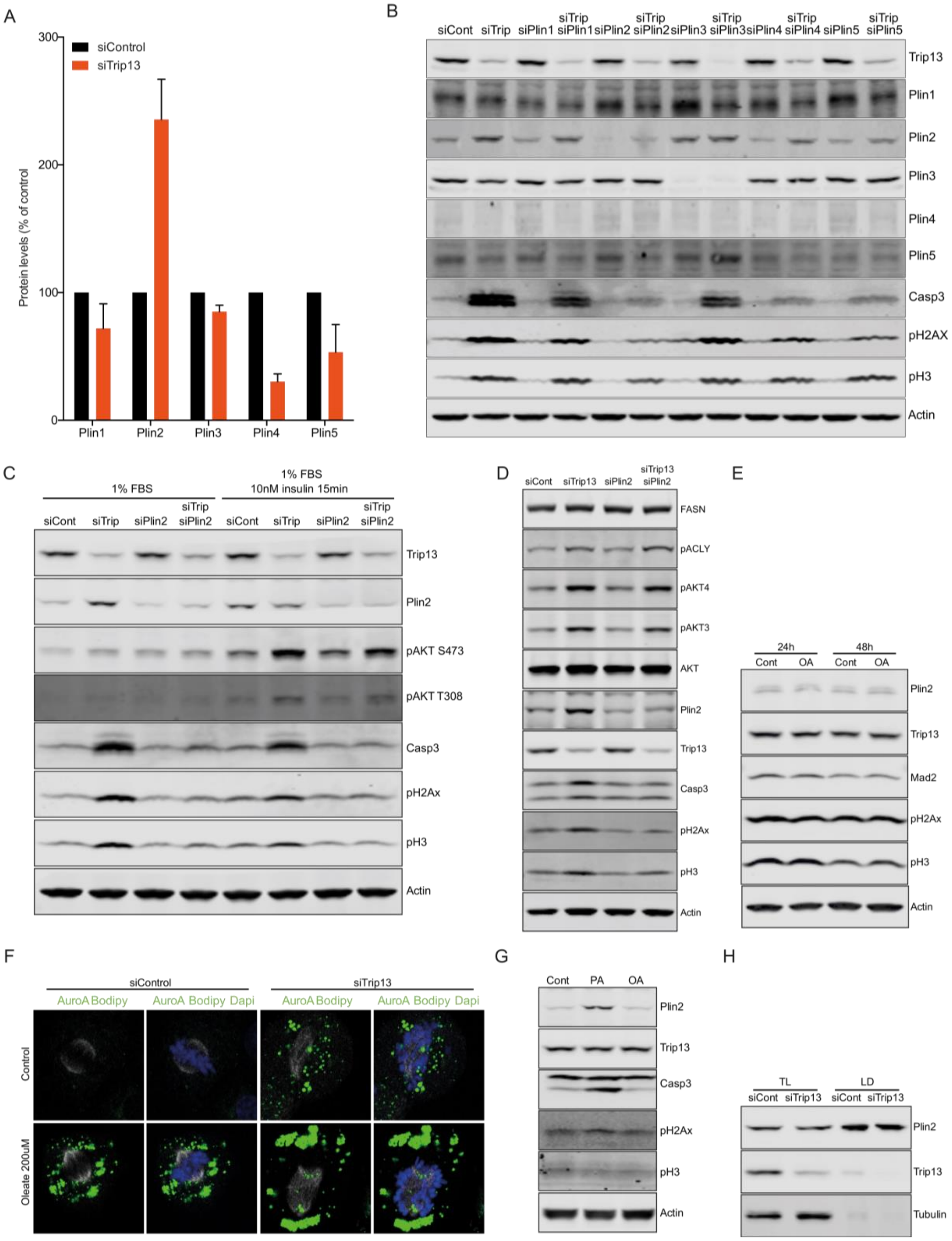

Figure S5. The lipid droplet coat protein perilipin-2 is a key component of the Trip13 KD-induced mitotic phenotype. A) Western blot densitometry quantification showing levels of perilipins 1-5 in HLF Trip13 KD cells. B) Western blots showing levels of cleaved caspase3 pH2Ax and pH3 in HLF cells transfected with siTrip13 alone or in combination with siPlin1-5 (n=3). C) Western blot of HLF cells transfected with siTrip13 alone or in combination with siPlin2, showing AKT pathway activation under basal or 10nM insulin stimulation. D) Western blot of HepG2 cells transfected with siTrip13 alone or in combination with siPlin2,

showing levels of Plin2, pACLY, FASN and AKT activation. E) Western blot of HLF cells treated with 200uM oleate for 24h or 48h, showing pH2Ax and pH3. F) Representative images of HLF control cells or upon Trip13 under basal or 200uM oleate treatment showing lipid droplets size and distribution. G) Western blot of HLF cells treated with 200uM palmitate or 200uM oleate for 48h showing levels of apoptosis, DNA damage and mitosis markers. H) Western blot of 200uM oleate treated HLF control or Trip13 KD total lysate or lipid droplet fraction showing Plin2 levels.

Figure S6

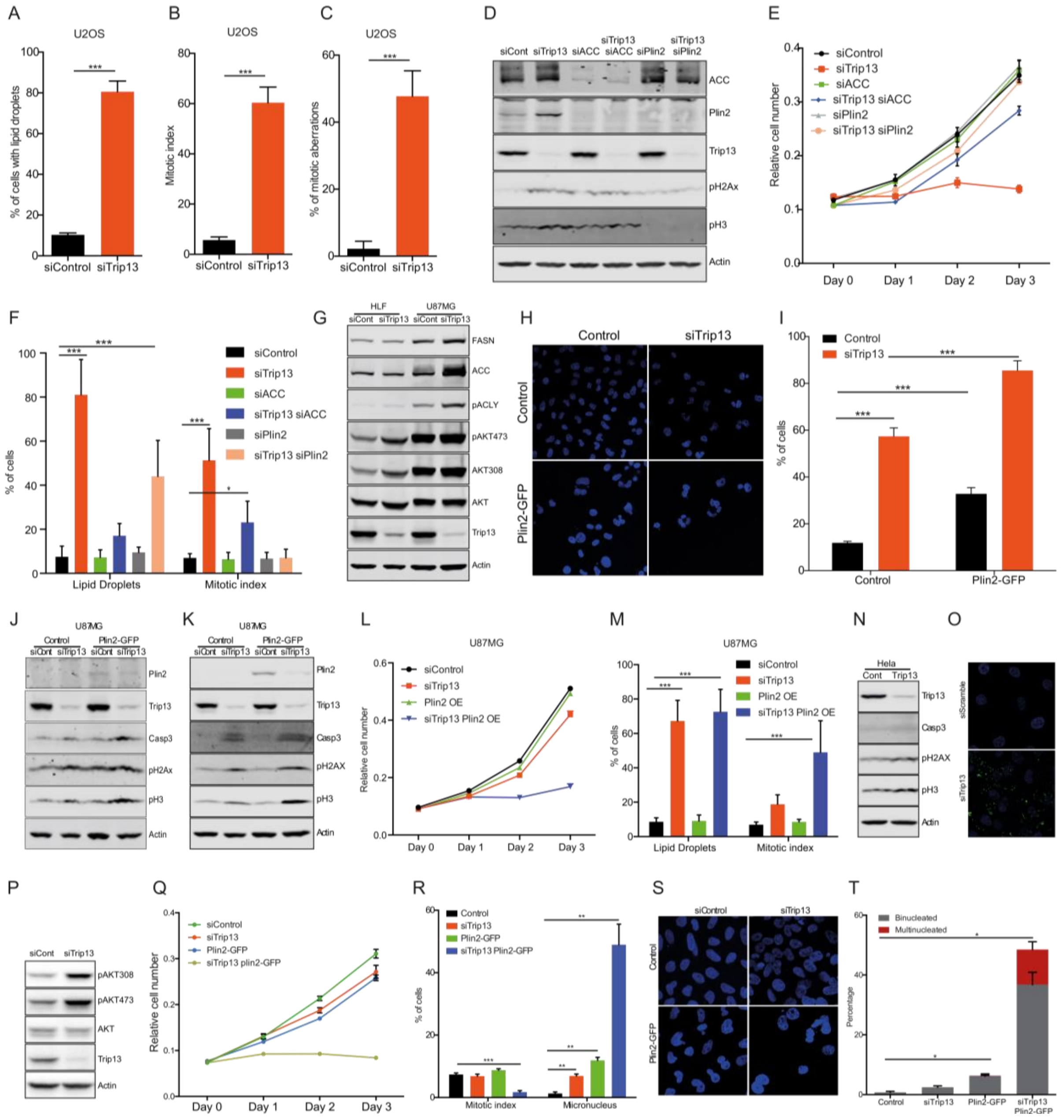

Figure S6. Perilipin-2 confers susceptibility to Trip13 KD-induced cell death. A) Lipid droplets, B) mitotic index and C) mitotic aberrations in Trip13 KD U2OS cells (n=3). D) Western blots showing levels of pH2Ax and pH3 in Trip13 KD alone or in combination with siACC or siPlin2 in U2OS cells. E) Growth curve of U2OS cells transfected as in D. F) Lipid droplets and mitotic index in U2OS cells transfected as in D. G) Western blots showing levels of pACLY, ACC, FASN and AKT activation in HLF or U87MG cells under Trip13 KD. H) Representative images of HLF control or Plin2 overexpressing cells alone or in combination with Trip13 KD showing interphase nucleus. I) Quantification of nuclear abnormalities from cells transfected as in H. J) Western blot of U87MG cells transfected with siTrip13 alone or in combination with Plin2 overexpression. K) Western blot of Trip13 KD in U87MG wt or Plin2 lentivirus transduced cells. L) Growth curve and M) lipid droplets and mitotic index in U87MG wt or Plin2 overexpressing cells alone or in combination with Trip13 KD. N) Western blot of HeLa cells KD for Trip13 showing levels of apoptosis, DNA damage and mitotic markers. O) Representative images of HeLa cells KD for Trip13 showing lipid

droplets in interphase. P) Western blot of Hela cells KD for Trip13 showing levels of AKT activation. Q) Growth curve of HeLa wt or Plin2 overexpressing cells alone or in combination with Trip13 KD. R) Mitotic index and percentage of micro-nucleated HeLa wt or Plin2 overexpressing cells alone or in combination with Trip13 KD. S) Representative images of HeLa cells transfected as in O showing interphase nucleus. T) Quantification of bi- and multi-nucleated HeLa cells transfected as in O (n=3). All data are shown as the mean ± s.e.m. n numbers refer to biological replicates. (F, I, M, R, S, T) 1-way ANOVA with Tukey's Multiple Comparison Posttest. (A, B, C) Student's t-test. *$P < 0.05$, **$P < 0.01$, ***$P < 0.001$.

Figure S7

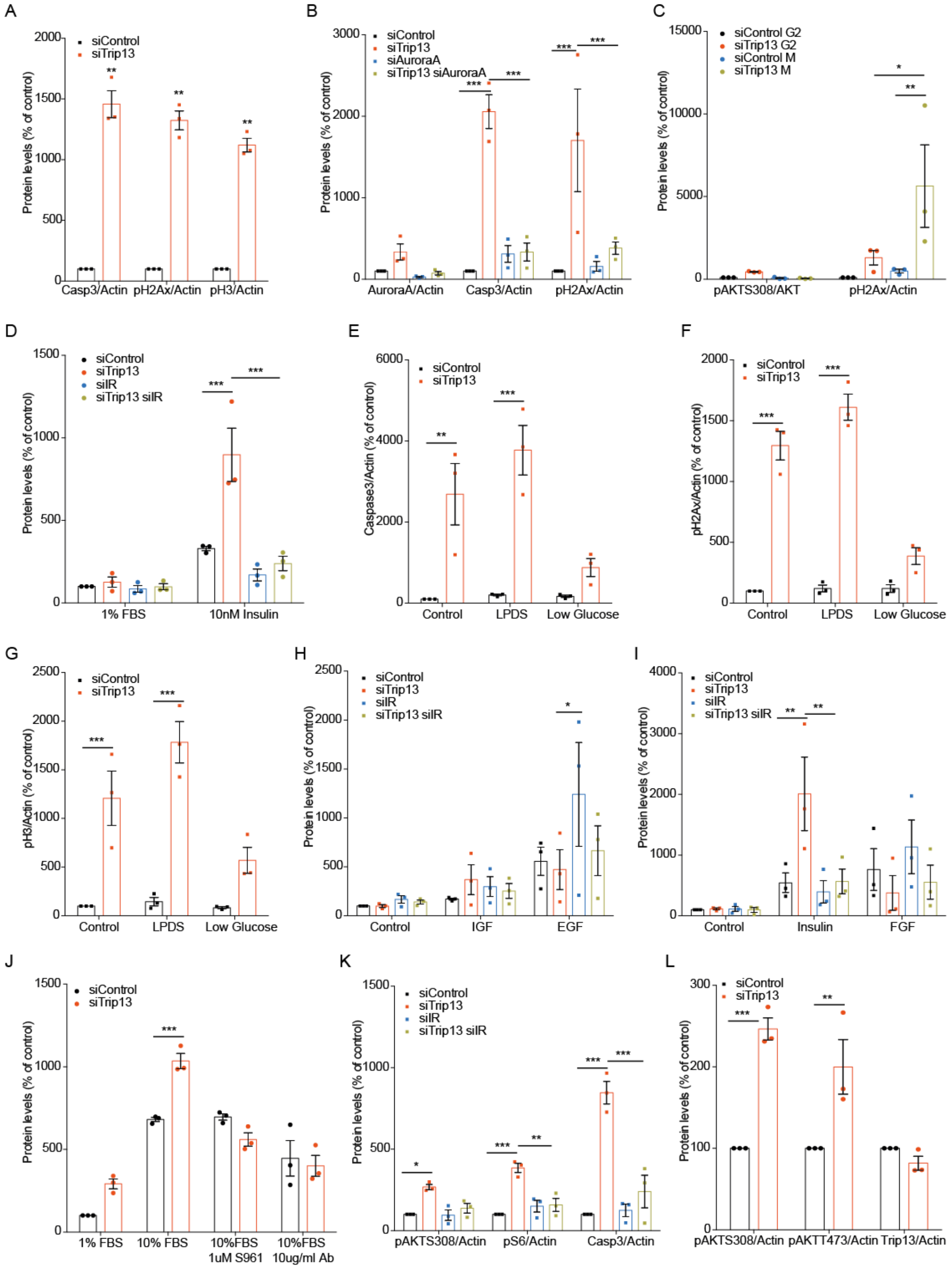

Figure 7. Western blot quantifications: A) Related to Figure 1F. B) Related to Figure 2 J. C) Related to Figure 3D. D) Related to Figure 3E and 3F. E) Related to Figure S3H. F) Related to Figure S3H. G) Related to Figure S3H. H) Related to Figure 3G. I) Related to Figure S3J. J) Related to Figure 3H and S3K. K) Related to Figure 3I L) Related to Figure S3R. All figures shown the quantification of 3 independent experiments as mean ± s.e.m. (B, C, D, E, F, G, H, I, J, K) 1-way ANOVA with Tukey's Multiple Comparison Posttest. (A, L) Student's t-test. *P<0.05. **P<0.01, ***P<0.001.

Figure S8

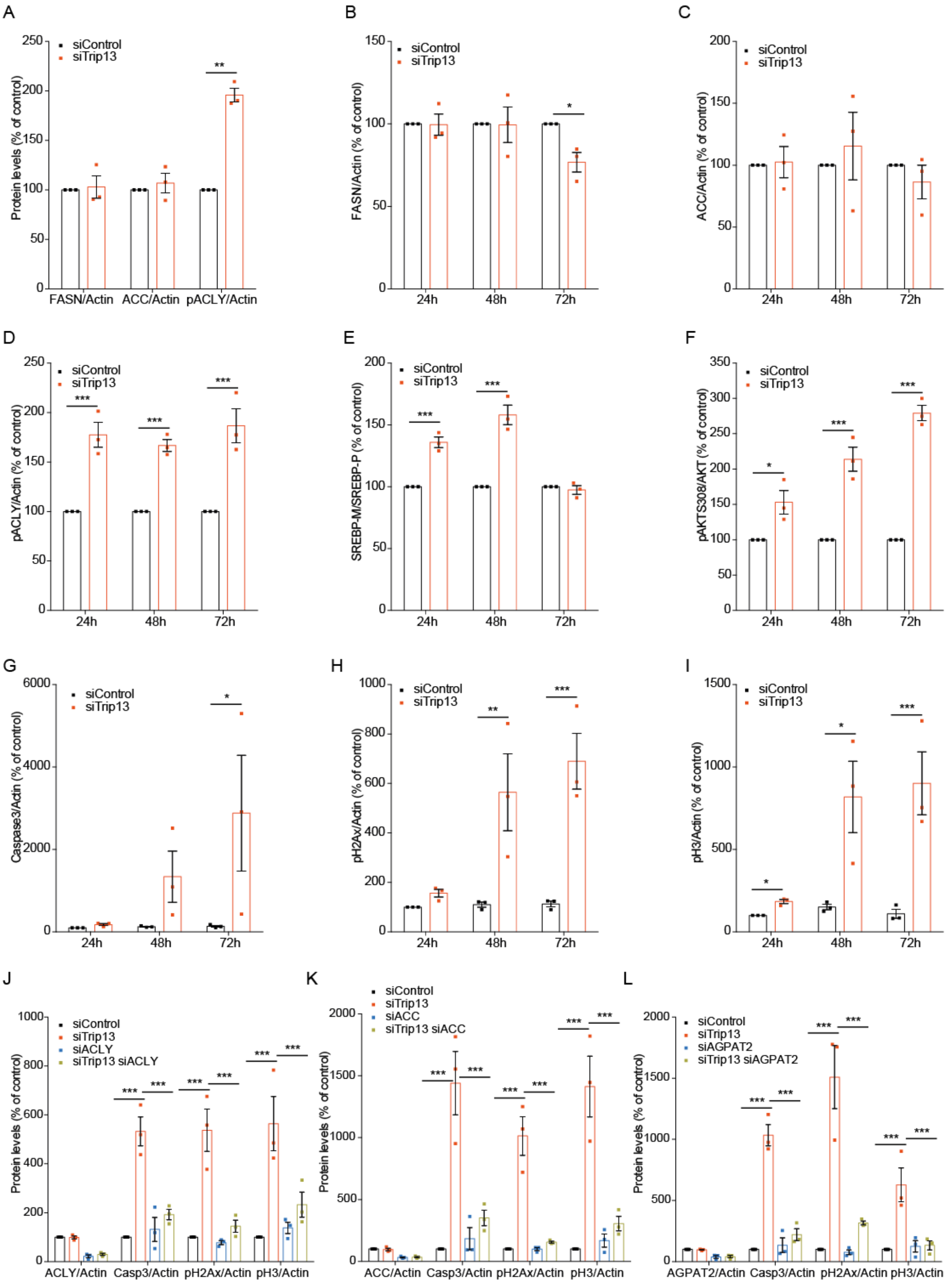

Figure S8. Western blot quantifications: A) Related to Figure 4F. B),C), D), E), F), G), H) and I) Related to Figure S4A. J) Related to Figure 4G. K) Related to Figure S4F. L) Related to Figure S4H. All figures shown the quantification of 3 independent experiments as mean ± s.e.m. (B, C, D, E, F, G, H, I, J, K, L) 1-way ANOVA with Tukey's Multiple Comparison Posttest. (A) Student's t-test. *P<0.05. **P<0.01, ***P<0.001.

Figure S9

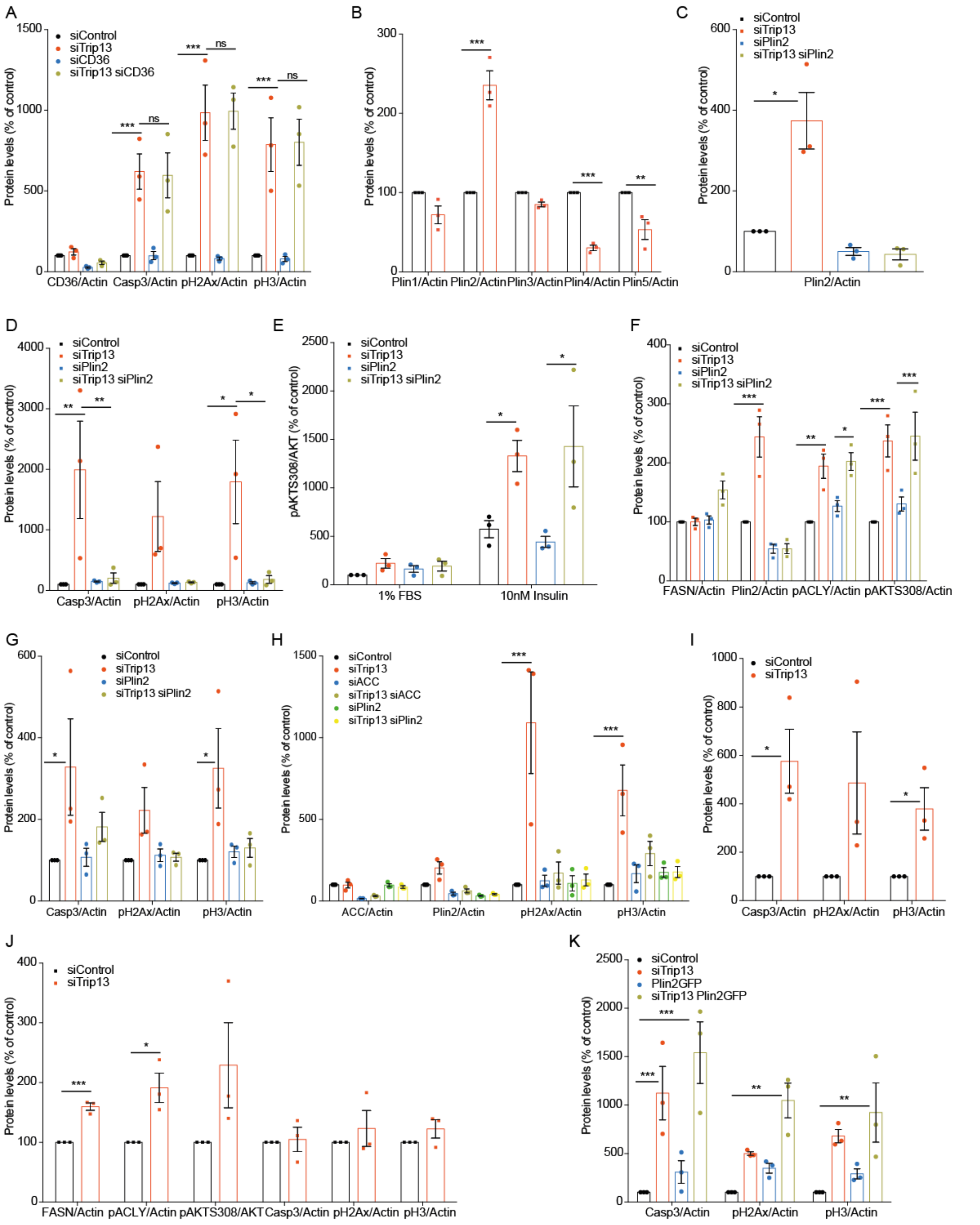

Figure S9. Western blot quantifications: A) Related to Figure S4J. B) Related to Figure 5A. C) Related to Figure 5E. D) Related to Figure 5E. E) Related to Figure S5C. F) Related to Figure S5D. G) Related to Figure S5D. H) Related to Figure 6A and S6D. I) Related to Figure 6B. J) Related to Figure 6F and S6G. K) Related to Figure 6G. All figures shown the quantification of 3 independent experiments as mean ± s.e.m. (A, C, D, E, F, G, H, I, K, L) 1-way ANOVA with Tukey's Multiple Comparison Posttest. (B, J) Student's t-test. *P<0.05. **P<0.01, ***P<0.001.

Figure S10

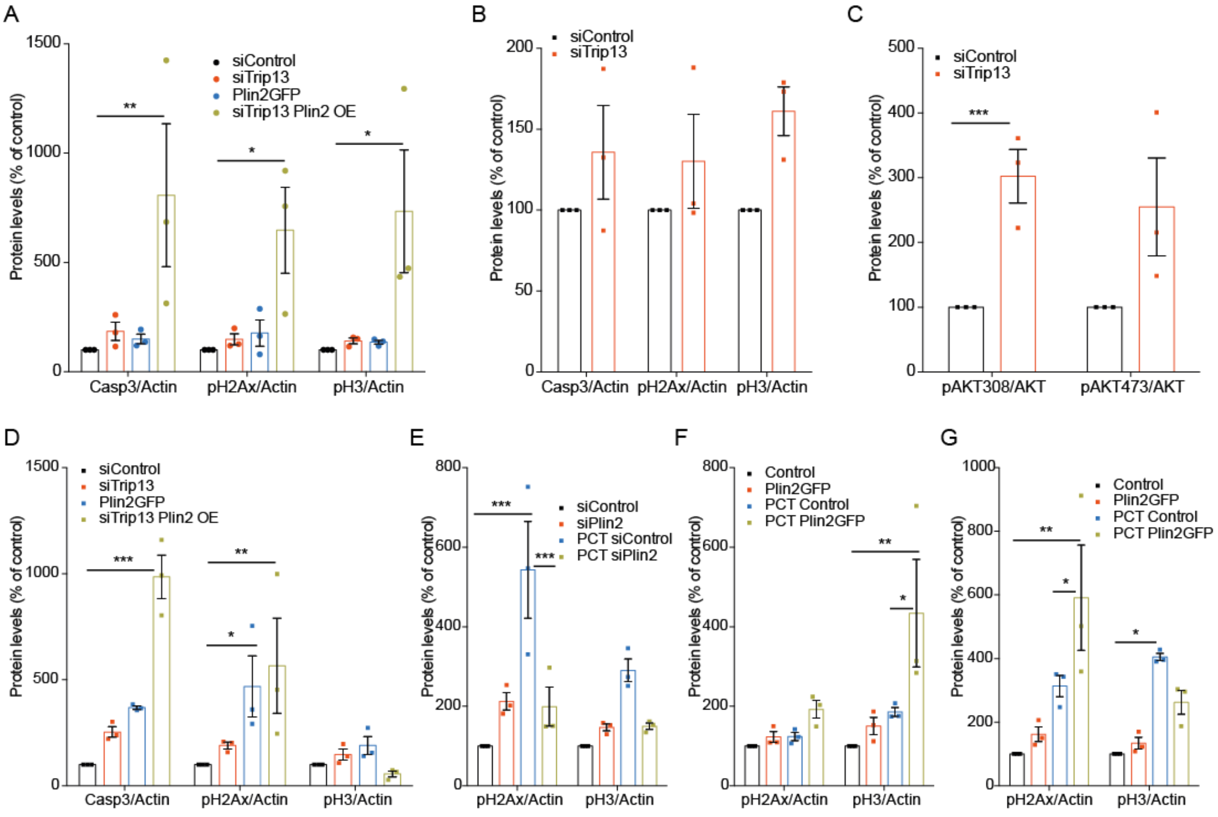

Figure S10. Western blot quantifications: A) Related to Figure S6J and S6K. B) Related to Figure 6L and S6N. C) Related to Figure S6P. D) Related to Figure 6N. E) Related to Figure 7A. F) Related to Figure 7C. G) Related to Figure 7E. All figures shown the quantification of 3 independent experiments as mean ± s.e.m. (A, D, E, F, G) 1-way ANOVA with Tukey's Multiple Comparison Posttest. (B, C) Student's t-test. $^*P<0.05$. $^{**}P<0.01$, $^{***}P<0.001$.

Supporting Information Movie 1 and 2. Related to Figure 4 and S4. Prevention of Trip13 KD-induced accumulation of lipid droplets rescues mitotic abnormalities and cell death.

Movie 1 (separate file). Representative time lapse of HLF unsynchronized cells constitutively expressing Aurora kinase A-GFP and H2B-mChrerry and transfected with sicontrol at day 2 post-transfection.

Movie 2. (separate file). Representative time lapse of HLF unsynchronized cells constitutively expressing Aurora kinase A-GFP and H2B-mChrerry and transfected with siTrip13 at day 2 post-transfection.
